# Spirit of the Foreign Periodicals, &c.

**Published:** 1843-04-01

**Authors:** 


### Spirit of the Foreign Periodicals.

### M. Forget's Clinique at Strasbourg.

M. *Forget* may be considered as the *Bouillaud* of the provinces. He is evidently full of intelligence and professional activity, and, although still under the dominion of his Parisian education, will, we think, ere long emancipate himself from many of his prejudicies *en ite.* This is pretty apparent from the tone in several passages of the annual report of his practice at the Strasbourg Hospital, published a few months ago. The very first extract shews that he is wavering in his ideas on the *enteric* doctrine of fever.

"In the present state of medical science, it is sometimes impossible clearly to distinguish simple irritation not only from mere disorder (embarras) of the stomach, but also from incipient follicular enteritis, or from an impending eruptive fever. It is the result of the case that determines the nature of the malady; the success of the treatment employed proves, by itself alone, little or nothing. We have however, in our practice, conformed to the precepts of the classical authors, by administering emetics and purgatives to those patients who were affected with the symptoms of *gastricity.* (such a word!) Many were relieved by their use; but it was not the less obvious that evacuants are generally very ineffectual in rectifying the state of the tongue."

The Professor gladly avails himself of the authority of *Stoll,* "le grand humoriste," to prove that, be the nature of a disease what it may, whenever any inflammatory symptoms are present, these must be first attended to. What rational physician ever doubted the propriety of the precept? but then we have never heard that *Stoll* or any other of the older authors, whom M. *Forget* is now so fond of quoting, recommends the use of antiphlogistic remedies when no symptoms of inflammation exist.

To proceed: there were 18 cases of "affections gastro-intestinales" treated during the course of the year. "Several of these," we are told, "might have been considered as cases of mild typhoid fever, if we were less severe in the qualification of diseases."

[Would that these Frenchmen used more intelligible common-sense language: they seem always to be acting on the principle of their wily countryman, that "language was given to man to conceal his sentiments."]

There were 16 cases of *diarrheic enteritis,* several of which also we might, "avec un peu de bon vouloir," have classified among the typhoid fevers.

Seven of these cases proved fatal. In all of them, there were found, on dissection, decided traces of inflammation in the large intestines. M. *Forget*'s treatment—and it seems, as well as we can judge from the imperfect report, to have been on the whole judicious—consisted in the use of antiphlogistics, in the first instance, then of opiates, and lastly of astringents: "such," he adds, "was the practice of *Sydenham*—with this one exception, that he employed purgatives, acting on his theory of the presence of acrid matter in the bowels." With a good deal of inconsistency, the Professor himself remarks, only a few lines afterwards, that "there are cases where mild evacuants may be serviceable, and others where the feebleness of the patient and the duration of the malady may forbid the use of anything debilitating."

We come now to a complaint about which the French writers have been bothering themselves a good deal of late—gastralgia, the diagnosis of which is, as we are told by M. *Forget,* "une chose grave et delicate." Unwilling to *go the whole hog,* in assenting either to the inflammatory or to the non-inflammatory doctrine as to the nature of this disease, he very wisely adopts a *juste-milieu* course, and avoids committing himself in any way.

The following sentence, however, looks a good deal like an abjuration of the Broussaian creed: "God forbid that we should deny the exaggerations of a fallen doctrine; (!) we fear, however, that the enthusiasm of opposition may carry many medical men into the very opposite errors. For our own part we have often successfully combated by means of a mild regimen, and gentle soothing medicines, several accidents caused by the employment of potent remedies, administered for the relief of supposed nervous affections." A sensible and useful remark; for, unquestionably, the use of stimulants and antispasmodics is often carried a great deal too far, and positive harm is done. Still, we have no hesitation in saying that very few cases of genuine gastralgia have any thing truly inflammatory in their nature.

During the year, no fewer than eight cases—of which five proved fatal—of cancer of the stomach were received into the hospital. (Is this disease more common in France than in this country?)

One of the fatal cases is so interesting, that we shall give an account of it at some length.

*Tumor in the Abdomen; Encephaloid Disease of the Stomach and Omentum, mistaken for an Ovarian Enlargement.*—A woman, 36 years of age, came into the hospital for a large tumor situated in the hypogastric region; according to her report, it was only of two months' standing. Three physicians, who had seen her, pronounced the case to be one of ovarian cystic enlargement. The right lower extremity was somewhat anasarcous; the swelling leaned over to that side more than to the other. One day, the patient was suddenly seized with frequent vomiting of a dark-coloured matter, like chocolate or coffee-grounds.

It was then, for the first time, discovered that several "bosselures" were perceptible in the epigastric region; and the conclusion, therefore, was that a cancer of the stomach existed. From this date, there was always present more or less tenderness of the abdomen; occasionally the vomiting returned; the wasting of the body was rapid, although there was never any decided feverishness. Twenty days after the appearance of these last-named symptoms, she died during the act of vomiting.

*Dissection.*—On opening the abdominal cavity, a quantity of fetid yellowish serosity flowed out; the whole of the peritoneum appeared to be inflamed, especially towards the pelvis; the liver was very bulky, and exhibited numerous encephaloid tumors, in different stages of softening: the hard irregularities, which had been felt during life in the epigastrium, were from these tumors in the liver, and not from the stomach, as had been imagined.

This latter viscus was, however, much dilated and lengthened in a vertical direction, its pyloric extremity being buried, so to speak, in the tumor that existed in the hypogastric region. This tumor, that was as large as the head of a full-grown fœtus, was hard and uneven; it was formed by an encephaloid mass developed at the expense of the omentum, and completely surrounding the pylorus, which itself had become degenerated into a substance of a similar nature. At the point of junction between the tumor and the pylorus, there was found to be a large perforation, which opened directly into the cavity of the stomach.

"Here," says M. *Forget*, "we see the many chances of error that occasionally occur in the diagnosis of disease. A tumor in the abdomen is taken for an ovarian cyst, and it turns out to be a cancerous degeneration of the omentum; the knobby irregularities felt in the epigastric region are attributed to disease of the stomach, whereas they were altogether owing to a morbid condition of the liver.

" During life, it was supposed that the cardiac extremity of the stomach was the part chiefly affected; but it was sound, and the pylorus was thoroughly diseased. A perforation existed in the stomach; yet this was not suspected during life. The peritoneum, also, was inflamed, and yet there had been no symptoms to indicate the disease."

M. *Forget* remarks that in not a few cases of cancer of the stomach, the symptoms are so irregular and uncertain, that it is scarcely possible to come to an exact diagnosis during life. " There is not," says he, " a single classical principle relative to the diagnosis of the disease, which does not find some contradiction in the cases which I have just related."

Let us now hear what the Professor says of the cases of typhoid Fever which were admitted into the hospital. He commences his report thus.—

" In spite of the specious arguments and severe criticisms which have been directed against our doctrines, we persist in regarding the *follicular enteritis* as the essential, if not always the primitive, element in all these fevers. There are three elements to be considered in the history of the disease—the fever, the typhoid disposition, (appareil typhoide,) and the follicular enteritis. These three elements constitute grave or malignant fevers. Of them one only, the last, is present in all cases; the fever and the typhoid disposition are sometimes awanting. It is therefore the follicular enteritis, which must be regarded as the most necessary and fundamental element of the disease in question. Such being our opinion, *we do not hesitate to classify typhoid fevers among the diseases of the digestive organs*—only with the distinction of grouping them together by themselves " en queue ou hors de ligne, " as deserving a special consideration.

Forty-four cases, in all, were admitted into the hospital, in the course of the twelvemonths; more of these occurred in Autumn and Winter than in the other seasons. The Professor strives to show from the circumstances of the residence, occupations, &c. of most of his patients, that miasms had little or nothing to do with the engendering of the disease. His reasoning on this point is very shallow, as may be at once seen from the following sentence, with which he closes his remarks: " before we can admit the influence of miasmatic poisoning, you must prove to us that the fever does never attack those persons who have not been exposed to it." If the learned Professor can tell us who are not exposed to such an agency, when it exists at all in any locality, we might admit the force of his reasoning; but certainly not till then.

Of the forty-four cases, nine only proved fatal. It is unnecessary to allude particularly to the favourable ones, as very many of them seem not to have been examples of typhus fever at all: no wonder, therefore, that they were quickly cured by the use of *emollients*. Let us briefly look to the fatal cases.

One man, who had just recovered from jaundice which had been treated with antiphlogistics and laxatives, was seized, a few days after leaving the hospital, with the symptoms of follicular enteritis. He was re-admitted on the 10th day after its *debut*, and was immediately bled from the arm. As the fever had a bilious character, the case was thought a favourable one for the use of purgatives. Considerable diarrhœa ensued; the typhoid state was aggravated; and the patient became delirious. Emollients, derivatives, astringents, and tonics, were successively employed, but all in vain; the patient died on the forty-first day after his reception into the hospital. On *dissection*, there were numerous ulcerations both in the large and in the small bowels. (Killed by the doctor.)

Here is another case. The report is translated literally. " A woman, antecedents not noted. Emollients, opiates, astringents, extract of nux vomica; against the diarrhœa, subnitrate of bismuth, enemata of nitrate of silver, tonics. Died on the 70th day. Generalised villous enteritis, some ulcerations; a few cicatrices."

(How gracefully brief and forcibly expressive!)

Without specifying other cases, may we not ask with some show of truth, is it not rather strange that no lesions seem to have been found any where else in almost any of the fatal cases, except in the bowels? Did M. *Forget* or his assistants look for them elsewhere?—we much doubt it. Did he, in any one instance, test the accuracy of M. *Andral's* recent researches on the state of the blood in fever? or was such a humoral notion as this deemed quite unworthy of the notice of a physiological physician? M. *Forget* seems anxious not to be taken for an out-and-out Sangrado doctor in his treatment of the disease; for, in summing up his observations, he takes care to tell us that three only of the patients were bled by his orders, and he reminds his readers that he has expressly declared, in his work on Follicular Enteritis, that "eclectism is the highest expression of the treatment of the disease." The concluding paragraph of his observations is so amusing that we cannot pass it over. "In our opinion the fundamental treatment is unquestionably the antiphlogistic; but this does not necessarily imply the use of bloodletting; for *expectation* forms part of it: (something like Paddy's definition of an 'armed neutrality'—a charge with the bayonet, to be sure!). In the present day a deplorable anarchy prevails in reference to the treatment of typhoid fevers; hence tonics and purgatives, as well as antiphlogistics have their zealous advocates. Heaven grant that the truth may ere long be found out; for it is this alone that we seek, even though it may convict us of error."

*Diseases of the Respiratory Organs.*

*Pringle* has very happily expressed an important truth in reference to the health of armies by this pithy expression, "plus occidit aer quam gladius." In Alsatia (the district in which Strasbourg is situated), as in almost every other country, diseases of the chest, induced by the vicissitudes of the weather, are the greatest scourge that we know, more especially among the lower orders of the people.

M. *Forget* alludes to the frequency of pulmonary emphysema being induced by, or at least associated with, chronic catarrh, and rendering the cure of it quite hopeless. The excessive use of expectorants, sedatives, &c. in many such cases often does a great deal of harm, by disordering the functions of the stomach; and such remedies can have but little effect on the disease. Quietude and outward irritation on the chest are the most important means of relief: opium, and especially the acetate of morphia, is often however a most necessary adjuvant.

The following remarks, on the use of the tartrate of antimony in inflammatory diseases of the chest, are very sensible.

"This medicine, exhibited in full doses, is in our opinion the second (bleeding being the first) of the heroic means to subdue such attacks. We almost invariably precede its use with bloodletting, if this can possibly be used;—partly because its good effects are thereby much promoted, and also because unpleasant effects have been occasionally observed, when the treatment was trusted to it alone. The less that a patient drinks, while taking the medicine, the better; as the *tolerance* is thus more readily induced. When this effect is once established, we may increase the dose of the medicine considerably each day. Three or four days are usually sufficient for the resolution of pneumonia treated in this way; and not unfrequently the favourable result takes place in 24 or 48 hours. As a general remark, we may state that we are not in the habit of using the tartrate except when the pneumonia resists bloodletting." (Would it not be wiser to use both remedies together, if each have an acknowledged efficacy on the progress of the disease?)

It seems that Pulmonary phthisis is a very frequent cause of mortality among the lower orders in Strasbourg. M. *Forget* is too honourable and too truthful a physician to pretend to cure this disease. In one of the fatal cases, a cavern was found on dissection towards the basis of the left lung, while the upper part of this viscus was quite sound. Such an occurrence proves the necessity of ausculting the whole extent of the chest, and not limiting the examination, as is too frequently done, to the upper part only.

Diseases of the heart also are very common in Strasbourg. We allude to them here only for the purpose of introducing the following remarks on cardiac auscultation. * * * "For a long time our attention has been directed to those interminable discussions on the causes of the cardiac sounds. To our view the theory of M. *Rouanet*—who attributes them entirely to the movements of the valves—is by far the most rational, and seems to be the one that accords best with physiological and pathological observations. According to it, the existence of any abnormal sound indicates the presence of valvular disorder; and seldom, it must be confessed, is this diagnosis untrue.

Aneurism or, more correctly speaking, dilatation with hypertrophe of the heart, is most frequently, but certainly not in all cases without exception, the result of chronic endocarditis (ossification of the valves with contraction of the arterial orifices); occasionally it seems to be produced by chronic pericarditis, or by any impediment to the free circulation of the blood, whether this impediment be situated near to, or at a distance from, the heart itself. Rheumatism is unquestionably a common cause of endocarditis; but certainly the two diseases are not so very frequently linked together, as alleged by M. *Bouillaud*: in not a few cases it will be found impossible to trace any connexion between them. M. *Forget* says nothing very satisfactory as respects the treatment of these cardiac cases. He omits to mention the most important of all remedies, a seton inserted over the heart. According to our experience, it is worth all other remedies put together.—(*Rev.*)

Under the head of *phlebitis,* we find some interesting remarks on the much disputed question, whether inflammation of the veins be a necessary antecedent of purulent infection of the system, and of the formation of what have been called *metastatic* abscesses in the viscera of the chest and abdomen. The result of M. *Forget's* observations hitherto has been rather unfavourable to the correctness of this theory. A woman was received into the hospital with severe tracheitis: the symptoms were so alarming as to threaten suffocation, and laryngotomy was performed with great relief to the patient. Subsequently however an abscess formed in the left hip, and all the symptoms of purulent resorption gradually came on. On dissection, several metastatic abscesses were found in the substance of the spleen, but none either in the lungs or in the liver.

In another case, one of albuminaria, a diffused phlegmon of the lower extremities supervened, caused by the scarifications that had been employed: the patient died in a typhoid state. In this case the spleen was the only viscus in which any abscesses were found. Now in neither of these cases could any traces of phlebitis be detected. "I have been long struck," says the Professor, "with the circumstance of the not unfrequent absence of any dangerous symptoms supervening on the spontaneous disappearance of outward abscesses, and, on the other hand, of the fatal consequences often produced by the absorption of the same fluid in cases of open wounds. In what other way can we account for such a difference, except by supposing that the purulent matter undergoes some unfavourable change by exposure to the air? For some years past, several cases of spontaneous phlebitis have occurred among our patients; and yet in scarcely a single instance do I remember that the symptoms of purulent absorption were manifested. A great deal depends, we are quite convinced, on the circumstance whether the purulent matter becomes exposed, or not, to the outward air." There has often been a good deal of unnecessary alarm on the subject of the supervention of phlebitis after venæsection. In not a few of the cases, in which an abscess has formed over the wound of the vein, the vessel itself is not at all affected; and in other cases, where there has certainly been a certain degree of phlebitis present, the disease has been readily checked by the use of appropriate means. Of these the most useful is unquestionably the application first of a number of leeches *above*, not *around*, the seat of the wound, and then of emollient poultices directly to it. I do not know, says our author, that the compression or the division of the affected vein above the seat of the disease has ever prevented the progress of the morbid action. If the symptoms of purulent resorption have once taken place, I know of no remedy that holds out the slightest prospect of doing good.

Under the head of *Alterations of the Blood*,* M. *Forget* ranks chlorosis, a disease which, he says, essentially depends on a diminution in the normal proportion of the fibrine and colouring matter of the circulating fluid. The researches of *Barruel, Lecanu, Andral* and others, leave no doubt on this point. Much importance has been attached by some writers to the existence of certain peculiar sounds that may be perceived by auscultation over the carotid and other large arteries in chlorotic patients, and some physicians have even gone so far as to regard the presence of these as the genuine diagnostic mark of the disease in question. Our author's opinion on the subject may be gathered from the following paragraph: "A useful sign, but one that is certainly very inconstant and obscure in its mechanism, has recently been insisted upon as the most expressive of all, particularly by M. *Bouillaud*—whose name alone would make us believe that the opinion was 'un peu outré'—*Rev.*—we allude to the blowing sound heard over the carotid arteries. We have heard this sound in all its shades and degrees, from the simple intermittent murmur to the continued *bruit de tempete*, and from the tick or buzz of an insect to the strong *bruit du diable*. These sounds may often be heard over other arteries and over the heart itself."

As a matter of course, steel is *the* remedy in most cases of chlorosis. The form in which it is exhibited does not matter a great deal, provided it is well borne and agrees with the system: M. *Forget* prefers pills made of equal parts of sulphate of iron and carbonate of potash. As to the lactate of iron and other preparations which have been recently introduced, they are all merely "affaires de mode." When the stomach is highly irritable, so that it does not bear the steel well, this may be combined with opium; and when the bowels are confined, with rhubarb. The following observations are very sensible: "It is much to be desired that all medical practitioners fully appreciated the spirit of the foregoing remarks respecting the *ætiology* of chlorosis. If such were the case, we should not see so much precious time wasted in endeavouring to bring back the catamenia, which very naturally often refuse to return for a length of time; or in trying to calm the palpitations of the heart by bleeding and such like remedies, which only aggravate the disorder; or in weakening patients who do not digest their food, by the use of strong purgatives, or lastly, calming by antispasmodic medicines obstinate nervous symptoms. Restore the blood to its healthy condition, and all these symptoms will vanish."—*Gazette Medicale de Strasbourg*.

---

### M. GIBERT'S LETTER ON THE LATE EPIDEMIC OF TYPHOID FEVER IN PARIS.

"I was present lately" writes this intelligent physician to his friend Dr. *Cayol*, "at a meeting of our hospital physicians, when various questions connected with

---

* The mere circumstance of such an expression as this in the writings of a Broussaian physician is a very emphatic sign of the change that is at the present time going on in medical doctrine throughout France. In a previous part of this Clinical Report, M. *Forget* remarks that "many affections, acute as well as chronic, are essentially derived from certain primary or secondary alterations of the circulating fluids"—an admission of the highest importance in a practical, as well as in a theoretical, point of view.

the reigning epidemic—during last Summer and Autumn in Paris—formed the chief topic of conversation; one of the gentlemen told us that, in a hospital which contains 230 beds, he had upwards of 60 cases of *typhoid fever* under his care at one time; another said that he also had had a great number, and had not lost a single case out of several hundreds; a third, communicated the important information that, provided no active treatment was employed, and the *expectant* method chiefly trusted to, nearly all the cases might do well; while a fourth added, that he thought mild laxatives in many instances useful. All agreed in denominating the disease as *typhoid fever*.

"But, although this name may have been brought into fashion by a school which is willingly satisfied with words in place of things, who is there but will not admit that the appellation is ill chosen at best, and that in particular it cannot be properly applied to the epidemic now prevailing in this metropolis? For in what does this epidemic really consist? In fevers induced by the high and continued heat of an unusually warm Summer, which has partaken in many respects of the character of a tropical season. Now these fevers (generally of a mild form, as already said) assume very rarely the proper *typhoid* character, but usually either a bilious, mucous or a catarrhal form; in some cases, the fever is inflammatory and continued; occasionally it is more or less remittent; and still more rarely has it anything of an adynamic or putrid character—the very character, be it observed, to which the term of *typhoid* would be least inapplicable.

"All these forms of febrile disease are evidently attributable to the mode of re-action in individual cases—a re-action, which manifests itself in different ways, according as the constitution of the patient is either sanguineous, bilious, or nervous.

"I have several patients at the present moment, in the Hospital St. Louis, affected more or less severely with the epidemic. In some it has been only an ephemeral fever, which passed off by sweating in the course of two or three days; while, in others, the fever has been of a catarrhal character; cough, nausea, slight diarrhœa, and a white coating of the tongue, being the most prominent symptoms. In one of the patients, the disease assumed the ataxic form, characterised by restlessness, delirium, paralysis of the bladder, and constipation of the bowels. In a few cases, the symptoms have been more or less remittent. Now I ask, is it not prudent to retain the old classic names of these various forms of fever, rather than to blend them all together under a single appellation, *typhoid?*—which, as far as the present epidemic is concerned, is unquestionably the least applicable and proper of all. Is there not a great practical advantage in retaining appellations, which in themselves suggest therapeutic indications? and has it not been clearly shewn that the pretended successes of certain statistic physicians, who have boasted of their having lost scarcely any patients under such and such a course of treatment, may be at once traced to the erroneous use of this phrase, *typhoid fever?*"

M. *Gibert* closes his remarks by stating that unquestionably the "medecine expectante" was, on the whole, the most safe and judicious mode of treating the late epidemic in Paris. In those cases, where the fever had somewhat of an ataxic or remittent character (usually without any shivering at the commencement of the paroxysm), he administered quinine with decided benefit; and in a few, where the vascular action was excessive, he had recourse to moderate depletion of blood.—*Revue Medicale.*

---

### M. Cayol's Letter on Typhoid Fever.

M. *Gibert* having requested the talented Editor of the Revue Medicale to communicate to the public his thoughts on this much vexed subject, he at once complied with the request of his friend, and the following extracts from his Letter will enable our readers to judge of the spirit and tone of his remarks.

"You ask me to talk with you on the subject of typhoid fever. Helas! you well know what I think of it: you wish me to put my hand into an open wound, to lay bare the miseries and expose the infirmities of the school that has been called the materialist, the anatomical, the anatomo-pathological, the physiological, the organic, the eclectic, or by what other epithet you may choose to know it."

.. .. .. .. .. .. "In medical science, there cannot be more than two schools, or systems of doctrine; and we may distinguish them thus:—

"The *first* views the organs of the body, whether in a healthy or a morbid state, as the instruments of life; the diseases as the abnormal re-actions or functions of the organism; and the organic alterations as the effects and eventual results of these abnormal re-actions or functions: we need scarcely add, that this is the vitalist or spiritualist school in medicine, and is the one to which we belong.

"The *second* seeks and pretends to discover in the organs or their contexture, in the molecules of which they consist, and in their material alterations, the reason, the *why* and the *wherefore* of life, and of all the physiological and pathological phenomena by which it is manifested: this is the anatomical or materialist school of medicine. This school, the superannuated daughter of the *philosophism* of last century, has no longer any point of support in modern science, which has shaken itself free from the material fetters, with which it was encumbered, to soar to a more elevated and intellectual sphere; but it now rests upon a spirit of *coterie*, and mutual admiration, which has succeeded marvellously well of late years. Adroit in profiting by all political circumstances, on every occasion, it has got itself installed into all the professional chairs, and into almost every scientific position that it can possibly reach; and thus it has acquired a sort of monopoly in the public teaching of medicine, most zealously excluding every one, who does not hold the same opinions, from having any share along with it. Every work, that is purely intellectual, is displeasing to it, and will often excite a movement of repulsion on its part; for it knows nothing and appreciates nothing but figures and material phenomena.

"As long as the anatomical school had a head, poised on the broad shoulders of *Broussais*, it managed to keep itself up to the height or position of a doctrinal system. By pushing to the extremest limits the localization of diseases, and referring to a gastro-enteric irritation all the phenomena of essential or primitive fevers, the celebrated reformer solved (doubtless, to his own satisfaction and that of his immediate disciples,) the puzzling question as to the proximate cause of these diseases—the culminating point of every medical doctrine.

"When *Broussais* died, his school died with him; no head, no doctrine:—or, rather, the doctrine died first; for every one knows that *Broussais* survived by several years the death of his intellectual offspring. At the same time, there was a schism in the camp of the materialists. The present existing *coterie*, which had already began to bestir itself not a little, occupied the same position in the school of medicine which the *doctrinaires* do in that of politics—they have no doctrine, and yet they regard themselves as the sole adepts in government-craft.* It (the coterie) refused to acknowledge *Broussais* as its legitimate head; it threw off the appellation of *physiological*, and took that of *anatomo-pathological*, *organic* and *eclectic*. The famous doctrine of gastro-enteritis being the

---

* It is curious to notice the connexion between sides in politics, and some of the schools of art and literature in France.

The Hippocratic physicians are almost all ultra-royalists; the out-and-out Broussaists are to a man either republicans or Buonapartists; while the middle men, the doctrinaires, those of the *juste-milieu* party, are generally friendly to essential and invariable cause of fevers, was not admitted without reservation: this, therefore, became once more a vexed question in the schools.

"The embarrassment was great. M. *Andral*, who at that time was engaged in publishing a second edition of his Clinique Medicale, no longer knowing what to make of fevers, left them out altogether. '*The progress of science*,' said he, 'has induced me not to devote, as in the former edition, a special volume to fevers.' Singular *progress* that! a few such steps, and medical science would be down at Zero. 'Yet,' continued M. *Andral*, 'I have carefully preserved all the observations contained in that volume; but have now given them another place, arranging some of them in the chapter *on diseases of the abdomen*, and others in that *on diseases of the nervous centres*.'

"We clearly perceive by these timid and embarrassed remarks, that the young professor had retained a slice of the gastro-enteric system, since a section only of the volume of fevers in the first edition was transposed to the chapter on diseases of the abdomen. We see, also, that he attempted to stitch upon this slice of doctrine a fraction of another, of quite a different nature:

*Unus et alter assuitur pannus.*

"The term *gastro-enterite* was now seldom heard of, and it was therefore requisite to substitute another. One proposed *dothinenterite*, another *enterite folliculeuse*, a third *exantheme intestinal* and so forth; but none of these were pliant enough to suit all circumstances and to embrace every kind of fever, at all times and under all circumstances. A new phrase was therefore necessary; and it was resolved to call the disease not *typhus*, (as it used to be,) but *typhoid* fever; and subsequently, in order to get rid of the objectionable word 'fever,' it was denominated *typhoid disease*, or *affection*.

"The word typhus (τυφος) signifies stupor; and hence, *Hippocrates*, and many writers after him, employed it to designate a grave continued fever, of which stupor or oppression was a predominant symptom. To apply, therefore, the phrase *typhoid fever* or *disease* to all continued fevers alike, shews the utter confusion of ideas, and the absence of all sound medical doctrine in the schools of the present day.

"If there is to be any positive meaning attached to words, we may surely expect that a *typhoid* fever will bear some resemblance in its essential characters to a *typhus* fever. When we speak of the *varioloid* disease, we signify an exanthem which approaches more or less nearly in its symptoms to genuine *variola*. Why then not follow the same rule, when we make use of the words *typhoid* and *typhus?* If there be not any τυφος or stupor, why call the disease *typhoid?* and how can it have any resemblance to the fever the very name of which points to this, as its characteristic phenomenon?

---

Louis Philippe and the existing state of affairs. The first set clings closely to every thing that is hallowed by age, and venerable from association; they love to throw an air of spirituality around all the phenomena of living nature; and hence their favourite writers are such men as *Chateaubriand* and *Lamartine*.

The Broussaists are the antipodes of these; they are all more or less decided materialists in their general philosophy, and regard with especial admiration the bold originality of the modern Romancists, such as *Victor Hugo, Balzac*, &c. The eclectics, or the third party, are intermediate between the two; their philosophy is altogether of a calmer character than that of either; less enthusiastic and poetical than the former, they avoid the cold abstractions and strivings after exactitude of the latter, who vainly seek to reduce every thing of living as well as of inanimate nature under the dominion of mathematical and arithmetical laws. Of this party, *Andral* is perhaps the head in medical, as *Guizot* is in political, science.

"Unable to characterise the fever, in a satisfactory manner, by any single anatomical fact, the gentlemen of this school have not the courage to characterise it openly by the expression of any vital phenomenon, as stupor; they are afraid of appearing too much of vitalists, if they did so; and therefore, as it would be rather ridiculous to give the name of *typhus* to a multitude of trifling febrile diseases, which no more resemble the genuine typhus fever than the bite of a flea does an attack of apoplexy, they have happily bethought themselves of the word 'typhoid'—a word which, expressing only a vague analogy, was supposed might be applied to all fevers, which they despaired of being able to characterise by any local lesions."

M. *Cayol* then criticises, with a most malevolent ingenuity, the work of M. *Louis*—entitled, "Anatomical, Pathological, and Therapeutic Researches on the Diseases known under the names of Typhoid, Putrid, Adynamic, Ataxic, Bilious and Mucous Fever, of Gastro-enteritis, Dothinenteritis, Follicular Enteritis, &c. compared with the most common Acute Diseases"—and exposes, with no less truth than severity, the egregious errors that the anatomical physicians of the present day have committed. "M. *Louis* has," he says, "with much more zeal than success, laboured to determine the exact necroscopic phenomena by which—(to use his own language)—*acute typhoid diseases* may be distinguished from *acute non-typhoid diseases.* Can any thing be more fanciful and arbitrary than this *à priori* division of diseases? Instead of taking his point of departure from something known and established, he assumes an imaginary line, and vainly hopes to solve a difficult problem by an insignificant and valueless word: certainly never was there a more complete confusion of ideas in a writer's brain than this. Who can believe that, in the category of *typhoid acute diseases,* M. *Louis* has not, perhaps quite unconsciously, admitted on the one hand some inflammatory diseases, and on the other hand some diseases that are not really typhoid? If any one could believe this, his mind will be at once disabused by reading the author's own observations; for we find from his work that in nineteen cases of *typhoid fever* there was found on dissection more or less decided splenisation or carnification of the lungs, that in several other cases tubercles or other pulmonary lesions existed, and in short that, out of the 45 cases of alleged fever that are described, these organs were sound only in 15, and in most of them there was some disease of the encephalon also at the same time. To take the other category of diseases, those which are arbitrarily designated as *non-typhoid,* it is possible to imagine that, among all the cases of peripneumonia, angina, scarlatina, apoplexy, and more especially still of protracted chronic disease, there were not many that had as much of a typhoid character as the contents of the other category?

"Let us now see what is the grand conclusion which M. *Louis* draws from all his arithmetical calculations and comparisons, touching the puzzling question at issue—the anatomical or material characteristic of typhoid fever. The answer which he gives is, that we find a morbid alteration of the "plaques elliptiques," (the glands of *Peyer*) of the small intestines in all patients who have died of *typhoid,* but not in those who have died of *non-typhoid* diseases. True, among many of the second class, we may meet with ulcerations in the intestines, and these ulcerations are even sometimes accompanied with swelling and redness of the mesenteric glands; but then, be it remembered, these ulcerations do not affect precisely the "plaques elliptiques;" and there lies the grand difference! Be careful not to confound in practice diseases of so very dissimilar a nature; the mistake might be most serious!

That it may not be supposed by any that we are at all wronging M. *Louis* by these remarks, we shall let him announce his discovery in his own words:—"The elliptic *plaques* of the small intestines having been found to exhibit a morbid alteration only in those persons who have died of the typhoid disease, and this alteration being constant, usually very serious, and always developed according to the same law, whether the course of the disease has been rapid or not, and in some cases also being the only lesion present, it is necessary not only to regard it as *peculiar to the typoid affection, but as constituting its anatomical character*, just as much as tubercles do that of phthisis, whatever may have been the cause that has induced their formation;" and in another part of his work he says, "in the present day it is now ascertained that all the different kinds of fever (the plague excepted) enumerated by *Pinel*, form only one and the same disease, the anatomical character of which consists, not in an inflammation of the stomach and intestines, (as maintained by *Broussais*,) but in a profound and specific lesion of the 'plaques elliptiques' of the small bowels. Except this peculiar alteration, all the lesions of the enteric mucous membrane, which may be found in typhoid fever, are observed after other acute diseases also; and indeed the frequency, with which they are met with in the two sets of cases, presents very little difference."

Having, as he supposes, determined this important point, M. *Louis* proceeds to discuss each symptom in the same manner of numerical analysis, and with the same minuteness of detail as he has already done with the various lesions of the different viscera found on dissection. "Four hundred mortal pages," says his redoubtable critic, "scarcely suffice him to register an infinite number of the most minute and wearisome details of pathological and semeiological facts—first isolated and by themselves, and then arranged in arithmetical tables, which can never either strike the mind or be fixed in the memory, from the utter absence of all logical cohesion between them. In such a manner as this, the observation of disease in the living body is more dry and barren than even that of necroscopic phenomena in the dead, as the one will not as readily yield itself to the isolation and arrangement of appearances as the other. Instead of having a series of living, and animated pictures, the study of diseases becomes nothing but a dead-letter."

M. *Cayol*, not satisfied with the attacks which he has already made on the anatomical theory of typhoid fever, returns once more, towards the close of his Letter, to the charge, and leaves M. *Louis* scarcely a foot to stand upon,—skilfully availing himself of the very weapons which the latter has put into his hands. "For the anatomical position, in respect to the cause of typhoid fever, to be true in the extended sense in which M. *Louis* insists upon, it is obviously quite necessary that the alleged alteration of the intestinal glands should be found in every fatal case of fever, from the most mild to the most malignant form; and also that it be not (generally at least) present in other diseases which are *non-typhoid*. This is what the author alleges to be the fact. Let us now see how the assertion will stand examination.

"In the first place, M. *Louis* himself, it will be observed, excepts the plague (the adeno-nervous fever of *Pinel*); and yet surely *it* is neither an acute phlegmasia, nor a simple eruptive disease. He excepts also from his catalogue of typhoid fevers not only the yellow fever of the West Indies and the epidemic cholera of the East, but also the 'typhus fever' of English writers, and the fever of camps and jails!—in short, the very fever that is the prototype of the disease, which he is vainly striving to discover and describe. Again, so sorely is he beset with the difficulties of his situation, that he is forced to admit the existence of a *latent* typhoid affection, to explain those cases in which the special alteration of the 'plaques elliptiques' is observed in patients who had not exhibited during life any of its usual symptoms, and also of a *simulated* typhoid lesion to explain other cases in which all these symptoms were present during life, and yet the special alteration of the 'plaques' was not discoverable on dissection—forgetting all the while that he had already committed himself by acknowledging that, 'if he ever came to observe a case in which all the symptoms now (actuellement) known of the typhoid fever existed, and where nevertheless after death the Peyerian glands were not diseased, he would not rank such a case among those of typhoid fever.'

"In a subsequent part of his work, when treating of an epidemic typhus fever which prevailed in Philadelphia in 1835, and in which the post-mortem examinations, performed with the greatest care, discovered no appearances of disease in the Peyerian or in the mesenteric glands, M. *Louis* is candid enough to declare that 'we cannot assign any seat to this disease, and that hitherto it has no anatomical character,' and he adds these remarkable words—'one feels oneself naturally led by the history of this epidemic to say, with M. *Valleix*, that the *typhus fever* may be really considered as *an essential fever*.' Oh! oh! M. *Louis;* you admit then that there may be such a thing as an essential fever! If so, what then becomes of your system of elliptic 'plaques,' and of your exposition of typhoid fever?

"After so many exceptions, so many contradictions, so many beggings of the very question at issue, is it at all necessary to adduce any other arguments to prove that the anatomical position laid down by M. *Louis* is certainly not correct, in the wide and general acceptation that he applies to it.

"But even if we admitted the truth of this very position, that the Peyerian glands are really more or less deeply diseased in all fevers, inflammatory, bilious, nervous, &c., we might ask, does it necessarily follow that all these fevers—differing, as we know that they do, from each other in their causes, their symptoms, the accidents which they are apt to produce, and in the indications of their treatment—must be one and the same disease? This indeed may be answered in the affirmative by the mere anatomist, who is only engaged with the dead body, and who does not watch the phenomena of the living one; but certainly not by the physician who studies the history and progress of diseases with a view to their treatment—unless indeed, after the example of M. *Louis*, he wholly rejects all the experience of the past, to make a *tabula rasa* of medicine, and re-commence *ab ovo* our science with the dissection of the 'plaques elliptiques' of the small intestines."

M. *Cayol* at length comes to the most important part of his commentary, that which bears upon the *treatment* of typhoid fever, as laid down in M. *Louis'* work. His remarks are altogether so admirable that we must give them in his own words:

"This part of the work occupies 120 pages, and consists of seven chapters, which treat successively of bloodletting, of purgatives, of opium, of tonics, of blisters, and of ice applied to the head. On each of these remedies, we have again and again new arithmetical data, and nothing more. A given number of patients have been bled; so many have been purged; while a third set has been treated with opium; and so on. We are then told how many recovered, how many died under each mode of treatment:—among the latter, what patients died on the 10th, or the 15th, or the 20th day; and among the former, what were the mild and what were the malignant cases.

"All this suggests the idea rather of a chemist operating upon a substance with a variety of re-agents, than of a physician giving the results of his clinical observations upon any disease in the living body. As might be foreseen, the arithmetical calculations of medical treatment have led the author himself to no positive conclusions. Indeed it would almost seem from his tables that the ratio of mortality is, *cæteris paribus*, nearly alike, whatever plan of treatment be followed! M. *Bouillaud's* celebrated system of bloodletting 'coup sur coup' does not reach a high mark in the numerical scale of his *confrere;* and as to other medications, nothing very positive is yet made out. We must wait for fresh figures and new tables."

Having thus shewn the utter fallacy of the mode of investigation pursued by the anatomical physicians of the present day, M. *Cayol* puts the very pithy question, what now becomes of *typhoid fever?* Nothing, but a senseless phrase, no better than the *peccant humour* of olden times. He strongly urges that it should be expunged from the vocabulary of medicine, unless an obvious and tangible meaning be affixed to it.

At the close of his letter, M. *Cayol* alludes to M. *Chomel's* work on typhoid fever in no very flattering terms: in his opinion, it is one of the very worst guides to be put into the hands of a young physician. It professes to be based on statistical facts; and yet many of these alleged facts are in flagrant opposition to the best-established truths. What shall we say of an author who lays down the following positions as the legitimate results of an extensive experience.

"A continued fever (the typhoid affection?) is never observed in persons beyond 40 years of life."

"It attacks a person only once during life. . . . The immunity is the consequence of a previous attack"

"It is never of shorter duration than 14 days."

"It is contagious, at least in the provinces."

"My pen," says M. *Cayol*, "falls from my hand when I have to transcribe such matter, and there is not a little of it in the book of which I am now speaking."—*Revue Medicale.*

---

### On the State of the Blood in Typhoid Fever.

During 1841, an epidemic Typhus prevailed in different districts of Italy. It was characterised by symptoms of unusually severe disturbance of the nervous system, as stupor, delirium, tetanus, convulsions, &c. Signor *Renzi* instituted an extensive series of researches to determine the state of the blood in the patients that were admitted into the Santa Maria Hospital at Naples. The field was an ample one, as it appears that between three hundred and four hundred patients were received in the course of the months of March, April and May. Being one of the physicians of the hospital, he fortunately availed himself of this favourable opportunity of testing the accuracy of the recent experiments of MM. *Andral* and *Gavarret* on the condition of the blood in fever—experiments which have excited no little stir among pathologists on the Continent, and of which we gave an ample summary in several of the late numbers of the Medico-Chirurgical Review. The conclusions, to which the Italian physician has come, go far to confirm the statements of his French brethren. He ascertained, for example—1, that the coagulum of the blood became soft and oleaginous: 2, that the proportion of its fibrine was very sensibly diminished from the standard of health; 3, that the proportion of the red globules was larger than usual; and 4, that the cruor was more or less mixed with the serum, being partially dissolved in it, tinging it of a red colour, and being precipitated in the form of a pulverulent sediment. The hematosine had but little coherence with the red globules and the fibrine.

Besides these observations already announced by the French pathologists, Signor *Renzi* has added some that are peculiar to himself. According to his experiments, it appears that not only was the proportion of the red globules increased above the ordinary standard in health, but the greater number of them, besides being readily freed of their colouring matter, seemed to have lost their central nucleus, and appeared, in consequence, to the eye of the observer, to be less compact, less solid, and, so to speak, less living than is normally the case. In the second place, there existed, according to our author's observations, in the blood of typhus patients, a peculiar smell, which was somewhat like that given out by the blood of a sheep just beginning to become putrescent.

The alterations now alluded to, were of almost constant occurrence in the numerous experiments of Signor *Renzi;* and yet he is too sensible a physician to jump at once to the conclusion, that the cause of typhoid fever resides solely in a change of the composition of the blood. Such a change cannot indeed fail to be regarded as the " point de depart" of a multitude of symptoms; but we must, at the same time, not fail to keep in remembrance the disorders of the nervous system—characterised by the stupor and the muscular prostration—and also the alterations of the mucous membrane of the intestines. In this point of view, typhus is considered—we need scarcely say, with perfect correctness—not as a mere local malady, but as a complex morbid state *sui generis*, quite distinct not only from any simple inflammatory, nervous, or cachectic disease; but also—and this is a most important feature in its history—from ordinary pyrexia (*synocha*, we presume the author alludes to). The alterations of the blood are so different in these two states (typhus and synocha), that this character alone is sufficient to raise up between them an insuperable barrier to their union.

From all these considerations it may be fairly deduced that every variety of the *typhoid affection* (such a stupid expression!), notwithstanding their diversity of type, has two common and constant signs—viz: a peculiar alteration of the blood, as described above, and a marked prostration of the energies of the nervous and muscular systems.

These researches on the alteration of the circulating fluid in typhus fever afford a satisfactory explanation of several of the phenomena which occur during its course. It is well known that M. *Magendie* found that, in proportion as he deprived the blood of living animals of its normal proportion of fibrine, so was the disposition to the occurrence of hæmorrhages and congestions increased. On this principle we may account for the dangerous tendency to visceral engorgement which often occurs in certain fevers, as well as to the effusions of blood from the capillaries in different parts of the system, external as well as internal. The petechiæ, vibices, &c., so often observed in the course of malignant typhus, are probably owing to what the old physicians called a dissolved (*defibrinated*) state of the blood.—*Il Filiatre Sebezio.*

We may here very appropriately introduce two or three paragraphs from some remarks by a M. *Facen* (in the Italian Journal " Memoriale sulla Medicina Contemporanea") on the state of the blood in Agues. According to his ob-observations, the blood, drawn at the commencement of an intermittent fever, scarcely, if at all, differs from its normal condition, unless the patient has been previously bled; and then the proportion of the serum is rather less than usual. The important alteration seems to be that, in almost all cases where the return of the paroxysms has been frequent, the tendency of the fibrine to coagulate firmly becomes greater and greater, until at length the blood acquires a perfect buffy crust, as in ordinary inflammatory diseases—the thickness and consistence of the crust varying, in different instances, according to the frequency, the severity, and the duration of the febrile paroxysms.

In accordance with the therapeutic indication afforded by this humoral change, M. *Facen* says, that the antiperiodic properties of cinchona are often very decidedly increased by the cotemporaneous use of antiphlogistic means.

---

### THE INFLUENCE OF ATMOSPHERIC AIR ON PUS.

It is a fact now well established that, if pus be exposed to the contact of the air, it soon acquires properties which render it strikingly deleterious, when it is conveyed into the torrent of the circulation, either by natural absorption or by direct injection into the veins. Healthy, or what used to be called *laudable*, pus may be injected into the veins of an animal without danger, provided the quantity be small; if too much be forced in, the pulmonary circulation seems to become mechanically obstructed, and the animal dies asphyxiated. When, however, purulent matter, that has become vitiated from exposure to the air, is introduced, a veritable infectious (we believe that the author means by this term rather *cachectic* than *contagious*) disease of the whole system is induced; the animal almost invariably dies; and on dissection the principal viscera are found to be the seat of purulent deposits, or what have been called *metastatic abscesses.*

M. *Bonnet*, chief surgeon of the Hôtel Dieu at Lyons, published a memoir in 1837 to prove that the symptoms of putridity, which are observed after injuries when the wound will not cicatrize, are owing to the absorption of sulphuretted hydrogen from the pus-discharging surface. No one can doubt that this gas is evolved, whenever animal matter becomes decomposed; and we daily witness its effects on the plaster used in strapping wounds; for this becomes black and discoloured in consequence of the action of the gas on the lead in its composition. There is however no good reason to believe that the small quantity of gas, that can be absorbed in the manner supposed by M. *Bonnet*, is the cause of the alarming constitutional symptoms, which not unfrequently supervene from the action of the atmospheric air on collections of purulent matter.

Other physiologists have suggested that another deleterious agent, hydrocyanic acid, may be generated during the process of suppuration; and they have supposed that the blue discolouration, which is not unfrequently noticed on the dressings of some sores, is owing to the presence of a cyanuret of iron in the discharge; but the recent experiments of Dr. *Conté* are certainly not favourable to this idea.

The cause of the fætor of unhealthy pus has long been a puzzle to the medical enquirer. To say that it is due to a vital agency is leaving the difficulty quite unexplained. Every one is now aware that purulent matter becomes changed at the expence, so to speak, of the constituents of the air; but hitherto not many experiments have been made to determine exactly the relations between the two. From the researches of Dr. *Conté* it seems to be clearly made out that the heat of the human body very decidedly promotes the decomposition of pus; for he found that it may be kept unchanged in a vessel for a much longer time than usual, if the temperature be low. (Perhaps a useful hint in the treatment of unhealthy abscesses may be derived from this circumstance: instead of using poultices and other warm applications, it may often be well to have recourse to cold lotions, &c.)

There is a certain local change in an ulcer itself which deserves more attention than it has hitherto received from surgeons—we allude to the inflammation of its surface and the destruction of the cicatrising membrane by the ammonia, which is apt to be generated under the dressings, and which is then kept confined by the strips of diachylon plaster that have been applied.

*Therapeutic Suggestion.*—Since the atmosphere may be so very injurious under some circumstances where organic products lodge in the tissues of the animal body, it might be well that surgeons would imitate the example of chemists in reference to certain substances, by keeping carefully protected from the agency of the disorganising element, the atmospheric air, parts that are inflamed and the products that are secreted from them. Surgeons have differed a good deal among themselves as to the best mode of opening many abscesses. Of late much attention has been drawn to this subject by the ingenious and very useful suggestions of M. *Guerin* in the department of what has been called "Subcutaneous Surgery." No one can doubt but this mode of exploring certain purulent collections is far more safe than that of making a direct incision into them. It is unnecessary however to pursue this subject further at the present moment.

### MELANCHOLY CASE OF SUDDEN DEATH, FROM THE INTRODUCTION OF AIR INTO THE VEIN.

A man, 58 years of age, was admitted into the hospital at Boulogne, with a large hard swelling, situated on the left side of the neck in the submaxillary region, extending downwards to within an inch or two of the clavicle. As it was believed to be of a scirrhous nature, the only chance of relief was pronounced to be from a surgical operation. A crucial incision was made through the integuments, and they were dissected back; then, partly with the scalpel, and partly with the fingers, the tumor was gradually detached from its connexions with the subjacent parts. The bloodvessels were secured, as they chanced to be divided. The operator now holding the tumor, which was adherent only by a narrow pedicle, in his left hand, and drawing it gently out, had made the last stroke with the scalpel, when all on a sudden he heard a peculiar noise, a sort of *glouglou* sound, extending from the seat of the wound in the direction of the region of the heart. At the same moment the patient became very pale, and his breathing hurried; he uttered a moaning cry, and said, 'I am dying:' and true it was; for, in a minute afterwards, he had ceased to live. Such was the terrible intantaneousness of the accident, that, although its nature was at once recognised, nothing could be done to prevent it.

The finger was at once applied upon the wound, and the chest was powerfully compressed, but all to no purpose.

*Dissection.*—In the lower part of the wound, the internal jugular vein, at about two *centimetres* distance from the subclavian, was found to have been opened: the aperture still gaped. By stroking the vein from below upwards, a quantity of blood mixed with bubbles of air escaped. When the thorax was opened, the lungs remained permanently expanded, so as to fill both sides of the chest completely. The right cavities of the heart were found distended: they collapsed by compression, and at the same time there escaped from the opening in the subclavian vein blood mixed with air. The auricle and ventricle of this side, on being opened, were found to contain a large quantity of bubbles of air mixed with fluid blood; and the same phenomenon was observed in the superior cava, and in the subclavian and axillary veins. The left ventricle was quite empty; there was neither air nor blood in it. The vessels even on the surface of the brain were found to contain, here and there, distinct bubbles of air.

The conclusions which the narrator draws from the statement of the case, are these:—

1. That the immediate cause of death was the wound of the internal jugular vein, and the admission, in consequence, of air into the circulation.

2. That the wound of this vein being kept open by the traction outwards of the tumor at the time, its proximity to the chest, and the enfeebled state of the patient—who had been much reduced by several attacks of hæmatemesis—all tended to favour the introduction of the air.

3. That, judging from the data furnished by the dissection, the suddenness of the death was attributable to the almost instantaneous cessation of the functions of circulation, respiration, and innervation.

4. That the introduction of the air into the circulation seems to have a special deleterious effect on the human subject; as, in none of the numerous experiments performed by MM. *Amussat*, *Nysten*, and *Magendie*, on lower animals, did death take place so rapidly as in the above case.—*L'Experience.*

In a recent number of the Annales de la Chirurgie, M. *Marchal* has made some remarks on the preceding case, and offered, at the same time, a new explanation of the cause of death in such accidents. That, in many cases, the death may be owing to the distention of the cavities of the heart and their consequent paralysis is very probable; but can we, in this manner, account for the almost instantaneousness of the event in other cases?—an instantaneousness almost as great as from a stroke of lightning, or from the injection of a drop of strong prussic acid into the veins of an animal. In such circumstances—which, be it remembered, have been observed only in the human subject, and never in experiments upon the lower animals—we are led to suspect that there must have been a toxic or directly-poisonous effect produced, just such as we find to follow the introduction of virulent matter into the circulation. M. *Marchal* suggests that carbonic acid may possibly be evolved, whenever atmospheric air is admitted into the cavities of the heart, and that it may be owing to the presence of this noxious gas that the rapidly fatal effects are owing. Why, he asks, should the blood on the right side of the heart, which is naturally dark, have become of a red colour?—unless some process of decarbonisation have taken place, the carbon having separated and become re-united with the oxygen of the introduced air.

There is a fact which appears to give some weight to this conjecture, and it certainly deserves notice—air, that has been respired, and which consequently contains an excess of carbonic acid, has been observed by many experimenters to act much more rapidly and fatally, when injected into the veins of animals, than unchanged atmospheric air.

I must not, however, conceal from myself, adds our author, that the experiments of *Nysten* with carbonic acid and with the oxyde of carbon, are certainly not favourable to the view which I have now suggested; but then, may there not be considerable difference in the results produced, according as the carbonic acid is directly introduced into the circulation, or as it becomes disengaged within the cardiac cavities? The question, it must be confessed, is still *sub judice.*

M. *Marchal* closes his remarks by a practical observation or two on the treatment of those frightful cases, when the air becomes admitted into the circulation, during the performance of an operation. He strongly inculcates the greater safety of breaking down the deep-seated connexions of a tumor in the neck with the fingers, or with any blunt instrument, than with a scalpel, and recommends that, in some cases, the tumor should be removed in different pieces rather than in its entire mass. Above all, when at length it is attached only by a narrow pedicle, let not the surgeon be in haste to divide this with the knife, if there be the slightest uncertainty as to the nature of the adhesions.

Had a ligature been previously passed round the pedicle, in the melancholy case related above, the accident might have been prevented: it was the last stroke of the bistoury that was followed by the frightful bubbling noise, and the almost immediate death of the patient. But is there nothing that a surgeon can do in such appalling circumstances, but to be the awe-struck spectator of the dreadful catastrophe? M. *Marchal* strongly advises the immediate employment of bloodletting: and if the blood cannot be obtained from a vein, let an artery be at once opened. He mentions that he has seen the good effects of this practice in several experiments upon horses, and, in an especial manner, on one occasion. The animal had fallen down senseless; a vein was opened, and the blood began to flow freely; in the course of a minute or two afterwards it started up, and scampered off across the field where the experiment was performed. M. *Nysten* has seen similar effects; and the hint is, therefore, not to be neglected.

---

### The Scientific and Medical Movement in Germany.

Nations, like individuals, have their illusions; they are apt to be blind to their own conduct, and also to be deceived in judging of that of others. In the eyes of most Frenchmen, a German is necessarily a man of fanciful imagination, an enemy of reality, fond of whatever is extraordinary, and not starting back even before the marvellous. This opinion is in part correct; but certainly not so absolutely, nor to such an extent, as is generally supposed. In truth, if we trace the progress of science in Germany, we shall speedily find how many striking refutations of this idea we shall meet with. In mathematical sciences, the land of *Euler* and *Leibnitz* has assuredly not degenerated in the present age; and the country, which still possesses such men as *Jacobi*, *Gauss*, and *Dirichet*, has nothing to fear from a comparison with others. Astronomy, unquestionably, is more cultivated beyond than on this side of the Rhine. Indefatigable observers may be found in every part of Germany, and many are the discoveries which have rewarded their labours. *Struve*, *Shumacher*, *Beer*, *Olbers*, *Bessel*, *Maedler* and *Littrów*, are esteemed the worthy successors of *Kepler* and *Copernicus*. In physical science, and more especially in chemistry, we have only to mention the names of *Berzelius*, *Rose*, *Mitscherlich*, and *Liebig*, to claim the highest rank for them. If we now examine the branch of medical research, which approaches most nearly to the exact sciences, we have occasion to remark a singular change that has of late years been going on in the progress of ideas, and in the direction of professional labours. At the commencement of the present century, the *philosophy of Nature* was in high estimation throughout Germany; and, under its influence, the anatomical writings of *Carus*, *Spix*, *Tiedemann*, *Meckel*, *Goethe*, &c. were composed—all shewing a marked tendency to a novel and daring spirit of generalisation. Different parts of the body were shewn to exhibit a somewhat similar type of organisation; and numerous relations, or marks of resemblances, between embryotic development and the structure of less highly organised beings, were pointed out with great ingenuity and skill. It has been to this spirit of inquiry that we owe the interesting researches of MM. *St. Hilaire*, *Serres*, *Blainville*, and others in France.

During the last ten years, a re-action seems to have been going on in this department of medical inquiry, throughout Germany. One might say, that its anatomists have been retiring back from the brilliant horizon that was opening up before them. While, in France, our philosophers have been extending the limits of transcendental anatomy, our neighbours have returned to minute descriptions, to delicate analyses, and to works of classification, which are the basis of comparative anatomy, but are certainly not its final end and aim. They heap materials upon materials, and have become as timid in their generalising deductions as their predecessors were hasty and daring.

In all this, we trace a tendency to the adoption of what has been called the positive spirit of inquiry. It is evident that, by confining themselves to the mere discovery of facts, and to simply forming such conclusions as obviously flow from comparing these facts together, there is certainly not much risk of being deceived; but then we can never hope in this way to arrive at the discovery of any of the general laws of anatomical science.

In medicine it is difficult to say what is the real tendency, among the German physicians at the present time. Two huge errors, Magnetism and Homœopathy, have issued from their schools; but neither of them has had much influence on the general progress of the science. In all places, and at all times, the sick and ailing will be found to be more or less superstitious; in their own eyes, their disease has always something unusual in it; and then it occurs to their minds, why should the remedy, necessary for its cure, not be so likewise? Little is the confidence usually inspired by the physician, who reasons with his patient, and tries to prove to him that he may often effect a cure of his own malady, by merely following the indications of common sense. Who is there so simple as to hope, that he will argue people of fashion into the belief, that a regular and temperate regimen is the principal adjuvant of sound health? and that it is often much more important to derive the means of cure from the kitchen than from the druggist's shop? The success of homœopathy and animal magnetism is based on the moral infirmity of mankind, and will just continue until another repast be provided for the jaded appetite of human credulity.

Let us not, however, be too severe upon our Transrhenal neighbours on this score; but let us rather tell them, in a spirit of kindness, that they have too generally shewn themselves tardy and unwilling to profit by the impulses which *Bichat, Pinel, Laennec,* and *Broussais* have successively given to medical science.

While everything has been sifted and examined among us, and every principle or doctrine, that has at any time been advanced, has been more or less frequently called in question, only to be the more accurately scrutinised; while effort upon effort has been made to re-construct the entire edifice of medical science, at one time by the aid of an improved classification, and at another by basing it on pathological anatomy, or on the localisation of diseases; while the French physicians have been engaged in the most animated controversies, and sparks and gleams of light have been constantly given out by the shock of opinions, our brethren in Germany have, with but few exceptions, persisted in the errors of a practice that is certainly not the most enlightened; wholly taken up with the study of mere symptoms without rising to the perception of the functional or organic lesions which give rise to them, they have always been striving to discover every minute secret of a system of therapeutics which at best is very problematical.

*Hufeland* may be considered as the representative of this epoch, and the naive assurance and air of confident satisfaction with which he lauds the remedies, which he employs in any particular case, make us only feel a regret that we cannot partake of a conviction that has been so easily acquired.

During the last few years however, a very decided re-action has been springing up among the medical men in some parts of Germany. The professors of the different universities, strong in their foreign languages, are making themselves well acquainted with all that is going on in other countries, and apply their minds with zeal to ascertain the truth of the novelties which they encounter in their reading. Pathological anatomy is cultivated with much zeal; large and beautiful works, on the model of that of *Cruveilhier,* have been published of late years; and at Vienna, more especially, Professor *Rokitansky* has given a strong impulse to the pursuit of this science.

We may consider Germany, in the present day, as the country where all the novel ideas and theories, which are broached in England and in France, are examined and discussed with calmness and unprejudiced patience.

No one system, no single opinion is dominant: all are received with impartiality indeed, but with caution and reserve. The humoral re-action, which is going on just now in our schools, will be received with favour by our neighbours; it is a return to the doctrines of the old medicine, a middle stage between the humorism of antiquity and the solidism of *Bichat* and his followers.

It thus appears that Germany is naturally called to judge between the various schools and systems of doctrine which divide French medicine; unless, indeed, there should appear one of those high and original geniuses, who, taking the start of their age, set all the world at rest for a time, because there is no one in a condition to oppose and overthrow their grand conceptions.—*Revue Medicale.*

---

### Case of Pelviotomy; Murderous Operation.

We observe, by some of the notices of Dr. *Lee*'s work on Clinical Midwifery (vide the last number of this Review,) in the Foreign Journals, that the French critics almost universally condemn the frequent use of craniotomy by British accoucheurs in many cases of difficult labour, where they would have recourse either to pelviotomy or to the Cæsarian section. If we sought for an additional argument in favour of British practice in this respect, where could there be a more convincing one than in the narrative of the following dreadful case?

A rickety woman, 23 years of age, was received into the Hospital for Incurables in Naples in the ninth month of pregnancy, and after labour had commenced. The membranes had partially given way two or three days before, and there had been occasional returns of pain ever since.

By measuring the dimensions of the pelvis with the pelvimeter of *Baudelocque*, it was found that the sacro-pubic diameter was about three inches: but, as the base of the sacrum was much inclined over to the left side, the space between this point and the sciatic tuberosity was less than an inch across, so that the left side of the outlet was entirely useless, so to speak, for delivery.

The Cæsarian operation being deemed to be too dangerous, in consequence of the length of time that had elapsed since the waters had began to escape, it was determined to have recourse to the performance of pelviotomy. The method pursued was that first proposed by M. *Galbiati*, and which consists in dividing, first, the rami of the pubic and ischiatic bones on each side, and then cutting the interpubic cartilage across along the median line. The operator, on the present occasion, was M. *Ippolyto*; but M. *Galbiati* himself was present. He (the latter) wished the operation to be deferred for a few hours, until the os uteri was more fully dilated than it was; but his opinion was over-ruled.

The steps of the operation are not related; all that we are told is, that it was very tedious, but well borne by the poor patient, and that there was very little loss of blood.

After the operation the woman was put into a warm bath; the expulsive pains, in consequence, became gradually stronger, but unfortunately the neck and mouth of the uterus remained only imperfectly dilated! The ergot of rye was therefore administered!! the pains increased, and at length, 20 hours after the operation, delivery *per vias naturales* of a full-sized child (which, however, unfortunately died during the labour,) took place!! Symptoms of peritonitis quickly ensued, and the poor woman died eight days afterwards.

*Dissection.*—The surface of the wound exhibited a gangrenous aspect; the peritoneal covering of the uterus was ecchymosed in several parts, and at one point there was a small quantity of purulent matter.

The comments by the editor of the Journal, in which this case is reported, deserve to be given here, as shewing the state of professional opinion in Italy, on one point of obstetrical practice.

"The operation was performed too soon. If it had been delayed until the os uteri was well dilated, the child might probably have been extracted alive immediately after the division of the pubis was effected.

By thus abridging the duration of the patient's sufferings, and sparing the uterus twenty hours of contractions, it is not unlikely that the mother's life, as well as that of the child, might have been saved."—*Il Filiatre Sebezio.*

*Remarks.*—The narrative of the case is exceedingly meagre; but this does not much matter as regards the only important question, "ought such an operation, as that now alluded to, ever to be had recourse to?" We should say, decidedly not. Perish, rather, a thousand children than that the life of one mother should be so inevitably sacrificed, as it must be, by such a murderous proceeding.

Just think of it; first to cut through and dissect the soft parts; next saw or nip across the rami of the pubis or ischium on one side; then do the same on the other side; and, lastly, divide the symphysis of the pubis; to add to all these horrors, it would seem that the labour is, after this dreadful suffering, to be left to be completed by the natural efforts of the uterus!!

We have read of many barbarous proposals, and still more barbarous doings, by surgeons of the present day; but certainly this novel obstetrical operation, which we observe is called *bipubiotomy*, surpasses all in outrage on science as well as on humanity. Heaven defend us from such a proposal being ever so much as entertained for a moment in this country! The man who presumed to do so, would be surely scouted either as a monster of barbarity, or as a madman.—(*Rev.*)

### On the Induction of Premature Labour.

The following observations by Professor *Stoltz* (of Strasbourg, we believe,) will be read with interest, more especially as they shew that a change is at work in the minds of many obstetrical physicians on the Continent in reference to this delicate and highly responsible point of practice—the induction of premature labour in certain cases of dangerous parturition. It cannot, indeed, fail to be gratifying, and certainly it is not a little creditable, to British accoucheurs, that they not only were the first most clearly to establish the safety and utility of the practice in question, but, for very many years, have unflinchingly persevered in their convictions of what is right, notwithstanding all the obloquy that has been cast upon them by their continental brethren.

Dr. *Stoltz* remarks, that the question under discussion "has now arrived at a complete solution," and he very candidly acknowledges that his countrymen have been the last to adopt the practice, and that it has not been without a good deal of resistance that they have at length yielded to the convincing arguments and proofs adduced by their opponents. He alludes to "the sudden change that has taken place in the sentiments of many leading accoucheurs in France during the last ten years, since the publication of M. *Burckhardt*'s Memoir at Strasbourg in 1830, and of his own paper, read before the Academy at Paris, in 1833;" and he then goes on to express his apprehensions, that his enthusiastic compatriots may—and indeed they have already began to do so—now carry their admiration of "this precious resource of our art," to as extravagant a length as they have hitherto done their condemnation of it. (How true of Monsieur Français on all occasions!)

"I have done my best," says he, "to prove the utility of the practice; I wish also to contribute to moderate the ardour of the enthusiasts, by shewing them the rocks on which they are apt to fall, and to convince them that the operation is one which requires much reflection, and no little professional sagacity. To attain this end, I intend to place along side of the reports of successful cases, those of others in which the practice has either failed, or been attended with serious consequences. The most perfect veracity and candour should be exhibited in the communication of all such facts: to deceive our fellow-practitioners on a matter of such high importance, is a scandal to the profession and a most grievous offence against humanity."

Before relating any new cases, Dr. *Stoltz* gives a brief account of the progress which the question has made in France since the publication of his Memoir in 1835.

M. *Banchereau*, a pupil of the faculty of Paris, defended in 1836 a Dissertation, entitled "On the Induction of Premature Labour in cases of Contraction of the Pelvis." It was, however, merely a copy of Dr. *Stoltz*'s Memoir: and the author neglected to mention that he (Dr. S.) was the first to perform the operation in France, and this at a period, too, when it was still considered as a crime by the law.

In 1837, there appeared a dissertation on the subject at Montpelier. Dr. *Figueira*, of Madeira, presented to the Faculty of Medicine in the months of this year, a work, entitled "Study of Artificial Premature Labour." At the close of his work, the author relates a case of Professor *Lovati*, taken from the review of the Obstetrical Hospital of Pavia in 1830-31.

In the course of the same year (1837) Professor *Villeneuve*, of Marseilles, published a report of the midwifery practice of the Maternity Hospital of that city, during the years 1835 and 1836, and in that report we find recorded a very interesting case, where premature labour was induced at the end of the eighth month in a rickety girl, whose pelvis was considerably deformed: a living child was born, and it survived at the time of the mother leaving the hospital.—(*Gazette Medicale*, 1837, p. 107).

"This," says Dr. *Stoltz*, "is the third case which belongs to France."

In 1840, M. *Caseaux* published his "Theoretical and Practical Treatise on the Art of Midwifery," and therein expressed his decided approval of the practice.

Professor *Moreau*, also, in his work (1840-41,) alludes to the question, and, without directly opposing the propriety of the practice, he surrounds it with numerous restrictions which very clearly prove, that the author is rather of the opinion of the school of *Baudelocque*, than of that of modern accoucheurs. Lastly M. *Chailly*, who has recently published a Treatise on Midwifery, expressly approves of the induction of premature labour in certain cases of difficulty, and admits that the operation has hitherto been resisted so long only from a foolish prejudice.

We may therefore, says Dr. *Stoltz*, safely affirm, that there are not many accoucheurs in France who still adhere to the doctrine of the *Baudelocque* school; but, on the contrary, that the induction of premature labour is very generally recognised by them, as a resource of the highest importance in the art of midwifery.

The change of opinion that has come over some medical jurists, in reference to this question, is rather amusing. One writer, who very peremptorily condemned the operation in the first edition of his work, pronounces a pompous eulogium upon it in the second; and Professor *Orfila*, who was one of the commissioners of the Academy that considered the operation as unwarrantable under any circumstances, has frankly acknowledged his error in the third edition of his Treatise on Legal Medicine, (1836, t. II. p. 347,) and now admits that "however plausible the arguments of M. *Capuron*, and others, may seem to be, there are unquestionably certain cases in which premature labour should be induced, in the hope of saving both mother and child."

---

### Obstetrical Cases by Baudelocque: Proposal to Tie the Internal Iliac Artery in Uterine Hæmorrhage: the Operation of Elytrotomy.

We have rarely read a medical paper that has astonished us so much as the one from which the following observations are taken. M. *Baudelocque* is nephew, we believe, of the famous Parisian accoucheur of that name, and is himself now a prominent professor of midwifery in the French Metropolis.

After some prefatory remarks on different forms of hæmorrhage from the uterus and vagina in pregnant women, he thus suggests the propriety of having recourse to a surgical operation, when other means have been tried without avail.

"I am of opinion," says he, "that it may be sometimes expedient to tie one of the internal iliac arteries, when the object of the accoucheur is to prevent, or to arrest, alarming hæmorrhage, whether this be arterial or venous, in the following cases:—

"1. In super-pelvic elytrotomy—(section of the vagina)—an operation which was the first, in France, to suggest in 1825, and which I performed on one woman during labour in 1829.

" 2. In some cases of extra-uterine pregnancy, to prevent the *arterial* hemorrhage which necessarily occurs, if the placenta be withdrawn immediately after the extraction of the fœtus.

" 3. In cases of sudden rupture of the vagina, in order to arrest the *venous* hæmorrhage, which is the immediate consequence of the accident.

" 4. Lastly, in certain cases of hæmorrhage, arising from the rupture of sanguineous tumors situated deep in the vagina—an accident against which the compression of the parietes of the vagina would be unavailing, and where even pressure upon the aorta could not be kept up long enough to arrest the loss of blood."

M. *Baudelocque*, having announced these extraordinary proposals, proceeds to describe the operation of what he has called *elytrotomy*, or section of the vagina from above the pubis.

" The usual Cæsarian operation," says, he, " having succeeded but in very few instances—in consequence, most probably, of the danger which always attends wounds of the peritoneum—it occurred to me in the course of the year 1825, that it would not be impossible to extract a child alive, and save the life of the mother too, in cases of deformity of the pelvis, by making an opening into one of the sides of the vagina from above, having previously made an incision through the tegumentary and muscular coverings in a line extending from the superior and anterior spinous process of the os ilii to about the middle of *Poupart*'s ligament, and then detached the peritoneum from its cellular adhesions to the internal iliac muscle. The operator may now feel the common iliac blood-vessels situated on the edge of the psoas muscle; and, as soon as he can distinguish the internal iliac artery and vein, he is to separate them, the one from the other, and pass a strong ligature round the former. The ligature being secured, the point of the scalpel is to be now plunged into the external wall of the vagina, as low as possible beneath the *ureter* (which is usually situated about a centimetre and a-half below the cervix of the uterus); the opening is to be enlarged with a probe-pointed bistoury, in a direction extending from the urinary bladder to the sacro-iliac synchondrosis.* This having been done, the operator now proceeds to extract the fœtus. If the head is found to present at the inlet of the pelvis in the usual position (supposing that the operation has been performed on the right side,) he will be able to lay hold of it with the forceps, ' et lui faire traverser, par la pointe du menton, l'incision faite au vagin.' It will be always possible to extract the fœtus by the incision in the vagina, if due care be taken to keep the small diameters of the head and trunk corresponding with the transverse diameter of this incision. The umbilical cord should not be divided until the child has begun to breathe. The placenta should be removed by the vagina, the cord having been previously passed back into it. This being effected, the two ends of the ligature round the internal iliac artery are to be

* That we may do no injustice to M. *Baudelocque* in describing this extraordinary operation, we shall give his own words, as we much fear that we do not quite understand all its steps. The external incisions having been made, the peritoneum exposed and separated from its adhesions to the iliac fossa, and the *internal* iliac artery being tied with a ligature consisting of several threads, " l'operateur enfonce la pointe du bistouri dans la paroi externe du vagin, en ayant la soin de le faire penetrer aussi bas que possible au-dessous de *l'uretere*, qui se trouve à *un centimetre et demi au-dessous du col uterin;* puis il aggrandit avec un bistouri boutonné d'avant en arriere, ou de la vessie à la symphyse sacro-iliaque correspondante, et de haut en bas, l'incision faite au vagin; puis il procede à l'extraction du fœtus."

passed into the vagina, and brought outwardly: the lips of the wound are then to be brought and retained together by strips of adhesive plaister, and with a proper bandage. When the patient is placed in bed, she should be instructed to lie on the side opposite to that on which the incision has been made, with the view of preventing the effusion of the lochia into the cellular tissue of the pelvis. M. *Baudelocque* adds, that it might be well that she should suckle her child for at least the first month.

(Really, on reading this last piece of advice, we were irresistibly reminded of Mrs. *Glass*'s very necessary instruction about making hare-soup, first to catch your hare:—first save your patient's life, say we, and that of her child too, and then it will be time enough to talk about suckling it.)

*Case* 1.—The first occasion that M. *Baudelocque* performed the operation of *supra-pubic elytrotomy* was in June, 1828.

The patient was a poor deformed, dwarfish creature, in the 36th year of her age, and at the full period of pregnancy with her first child. Labour had commenced; the os uteri was partially open, and there had been a free discharge of the waters for some time, before M. *Baudelocque* proceeded to perform the operation which we have described above. When the incision was made upon the walls of the vagina, (the internal iliac artery had not been tied,) a profuse hæmorrhage took place. In vain were all the attempts made to arrest the flow of blood; and, as the patient's strength seemed to be sinking, M. *Baudelocque* immediately performed the Cæsarian operation, and extracted a child that had recently died. The placenta was extracted by the wound in the abdomen, and this was afterwards brought together with six stitches.

A good deal of blood was lost upon making the incision through the uterus, and the hæmorrhage continued, although not in great quantity, after the patient had been placed in bed. "In one word," says M. *Baudelocque*, after some attempts at explanation, which it is unnecessary to re-produce "she died in consequence of the venous hæmorrhage from the divided vagina and uterus." (Is it not rather strange that M. B. does not mention how long his patient survived the two dreadful operations to which she had been subjected? Did she sink within a few hours of it?)

He goes on to say, that it would have been very useful, in this case, to have tied the internal iliac artery on the side on which the operation was performed, before the incision had been made into the vagina. "It is incontestable," he has the hardihood to add, "that by tying this artery, and waiting for a minute or two until the branches of the internal iliac vein were disengorged of their contents, the section of the vagina might have been made without the least loss of blood (?); and it may also be readily understood that, if instead of operating on the left side, where the vertebral column projected so much that there was not more than a centimetre and a-half between it and the os ilii, the operation had been performed on the right side, I should have been able to have extracted the fœtus with the forceps, although the head presented itself at the inlet of the pelvis in the first position."

Such are the presumptuous reflections with which the history of this most lamentable and disgraceful case is closed. We trust that it will be long ere we again meet with anything at all like it.

*Case* 2.—*Extra-uterine Pregnancy; Section of the Vagina and the Fallopian Tube; Extraction of a Fœtus; Sudden Death.*

A healthy and robust married woman, 38 years of age, who had never had any children, observed that, about the beginning of April 1831, her catamenia, which hitherto had been very regular, began to cease, and to be replaced by a scanty discharge of reddish serosity. Shortly afterwards, she had repeated attacks of vomiting, and, at the same time, experienced sharp pains in the hypogastrium, which gradually increased in fulness.

This state of things continued for about four months and a-half, at which time she thought that she could feel the movements of a child. M. *Reis* was first applied to; on examination *per vaginam,* he found a tumor on the left side of the uterus. *Dupuytren,* who was afterwards consulted, could not have understood the nature of the case at all, for he prescribed mercurial frictions on the abdomen.

The tumor could be distinctly felt through the abdominal parietes, and its size seemed to be nearly as large as that of the uterus itself at the supposed period of pregnancy. The belly gradually became fuller and fuller, and every now and then attacks of sharp pain, accompanied with vomiting, came on. On the 4th of Nov. I visited, says M. *Baudelocque,* the patient, along with M. *Reis,* and could distinctly feel that there were two tumors in the abdomen; the larger one being on the left side, rising up as high as the umbilicus, and occupying almost the entire extent of the hypogastric region. The right or smaller tumor was of a rounded form and seemed to be somehow attached to the other. The mammæ were, and, according to the woman's report, had always been, quite flaccid. On examination of the vagina, even when the entire hand was introduced, no trace of the os tincæ could be felt, although, some months before, M. *Reis* had felt it quite distinctly. M. *Baudelocque* was of opinion, from a consideration of all the symptoms, that the larger tumor was formed by the pregnant uterus, and the smaller one by an extra-uterine conception in the right Fallopian tube. A distinct *bruit de soufflet,* it may be mentioned, was perceptible on the left side of the hypogastrium. I proposed, says M. B., to free (debarasser) the woman, by making an incision through the vagina and the right Fallopian tube, for the purpose of extracting the child, which I deemed to be alive. Her parents requested that there should be a consultation with another physician previously: M. *Recamier* was fixed upon. After a very careful examination, he also was of opinion that there was an extra-uterine pregnancy, and that the child should be extracted by an operation. Next day, this was performed in the following manner:—The left hand having been slowly and gradually introduced into the vagina, I laid hold of the tumor supposed to be formed by the Fallopian tube, and passed a long narrow bistoury upwards, till its point reached the most projecting point of the swelling. The instrument was then plunged fairly into it; and forthwith a quantity of sanguinolent water flowed out from the vagina. Carrying my forefinger into the cavity of the tumor, I felt the fœtus distinctly, but could not determine the exact position in which it was placed. The incision having been enlarged by means of a blunt-pointed bistoury, I then endeavoured to apply the forceps to the projecting part of the child. Considerable difficulty was experienced in effecting this; but at length it was accomplished, and I succeeded in bringing the fœtus down to the vulva.

It was a hip-presentation; the lower extremities and trunk were extracted with tolerable ease, but it required not a little force to bring the head through. The operation lasted between thirty and forty minutes in all. The child was discoloured, and its limbs quite flaccid; it made one deep inspiration. M. *Recamier conferred baptism upon it.*

The sequel of the case is given in the following words:—

"Up to this time, the woman did not seem to suffer much more pain than what often occurs during a difficult labour; but, *unfortunately for her,* I peeled off (decollai) the placenta, or, to be more exact, I tore it away piecemeal (arrachai partie par partie,) not being able to bring it out entire. In proportion as I tore it away, a very copious hæmorrhage followed.

"To arrest this, strong pressure was made upon the abdomen, and plugs of charpie were introduced into the cavity, but without success. By the advice of M. *Recamier,* I passed an œsophageal tube well up, and injected cold water into the sac; but this, too, failed; and the woman, becoming gradually more and more exhausted, at last expired *quelques moments après l'arrachement du placenta.*"

On opening the abdomen, a large tumor, whose sides had partially collapsed, was found to extend fairly from one side to the other, and to fill up entirely the upper part of the pelvis. It was formed by the left Fallopian tube, which had become so much dilated upwards as to rise above the level of the umbilicus. The uterus, also, was considerably enlarged to about the size it usually is between the fourth and fifth months of pregnancy; its cavity was lined with a membranous covering, and in the substance of its fundus there was a fibrous tumor larger than a hen's egg.

It thus appears, that the diagnosis of the case, formed by MM. *Baudelocque* and *Recamier*, was not at all correct; and that the large swelling felt on the left side of the abdomen was from the dilated Fallopian tube and not from the uterus—in the cavity of which there was no product of conception at all, as had been imagined.

M. *Baudelocque* proceeds to state how he would act in another such case, as the lamentable one now recorded. It is unnecessary to follow him in his disquisition on this subject, as it seems to us, that any remarks of his are not likely to have much weight even with his own countrymen, far less with any British practitioner. As may almost be anticipated, he gives the preference to *his own operation* of elytrotomy over any other in cases of pregnancy within the Fallopian tube, as well as in those of impracticable labour from deformity of the pelvis.

*Case* 3.—The third case, reported in this memoir by M. *Baudelocque*, is not less deserving of attention than the former two, as giving us some insight into the practice of French accoucheurs in cases of difficult parturition. We therefore request our readers' attention to this report, in order that they may be able to determine for themselves the respective merits of the advices laid down in the works of British and of Continental writers on Midwifery in the present day.

M. *Baudelocque* was summoned by a brother practitioner to a case of shoulder-presentation, where repeated attempts to turn and extract the child had been already made. On passing his hand up the vagina, he found that the canal was lacerated (to what extent is not mentioned). As the uterus was contracting strongly at the time, he recommended that the woman should be first bled. This was done; and he then, but with much difficulty, effected the turning and delivery of the child. Two or three hours subsequently some very awkward symptoms—vomiting, great anxiety, rapid pulse, pallor of the face, and cold sweats—supervened.

The attendant practitioner, although evidently much alarmed, attributed them either to "un epuisement nerveux," or to the "introduction de l'air dans les veines de l'uterus." He wrote off immediately to M. *Baudelocque*, requesting him to state *what was his view of the case, what authors might be best consulted on the subject, and what treatment would be most expedient.* (!)

The woman died soon afterwards,

On dissection, a considerable extravasation of blood was found within the peritoneal cavity near the vaginal laceration, *which extended from the left side of the bladder to the sacro-iliac symphysis, and consequently involved the peritoneum.*

Although most British accoucheurs would decidedly disapprove of the practice pursued, even by M. *Baudelocque*—not to mention that of the other medical man—in the present instance, and would have had recourse at once to embryotomy and speedy extraction of the fœtus, instead of first lowering the woman's strength by a copious bleeding, (she being at the time exceedingly exhausted), and then turning the child when the vagina was lacerated, we really did intend to have passed this case over without any censuring comments, till we came to about the close of the report, and, reader, what do you suppose we found there? We must give the author's own words:—

"How much I regret that I had not sooner had the idea of tying the internal iliac artery! I should certainly have performed the operation on this patient. I should have made an incision in the left costo-iliac region, separated the peritoneum from the iliac fossa, and have tied the internal iliac artery, after having cleaned out (nettoyé) the peritoneal cavity of the blood which it contained; then, master of the venous hæmorrhage, I should have extracted the child through the laceration in the vagina, instead of turning it and bringing it out in the usual way, or, perhaps, I should have done better by dividing the neck of the fœtus, and then extracting the head either by the pelvis or by the wound in the vagina: at all events, I should avoid turning the child, in order not to be obliged to have recourse to bleeding. By acting in this manner, I might have prolonged, if not saved, the life of this patient, and then I should have treated her for the peritonitis, which must necessarily have supervened on the rupture of the vagina." (!)—*Journal des Connois. Medicales, Janvier* 1842.

---

### M. Civiale on Contractions of the Urethra.

The following letter was recently read before the Academy of Medicine:—

"I have the honour to submit a memoir in which I have examined the following three important questions—What are the morbid states which constitute urethral contractions? To what material or organic effects do they give rise, when they are neglected? What are the evils that may be occasioned by the therapeutic instructions contained in most surgical works?

"By collating the numerous facts recorded in different books with the results of my own experience, during an extensive practice for the last 20 years, and also with the observations I have recently made in my examination of the rich museums of London, I hope to be able to throw light upon some disputed points in the pathology of the urinary organs.

"1. It is generally imagined that the organic lesion, which constitutes the stricture, is an accidental formation developed on the internal surface of the urethra, and the removal or destruction of which is necessary for effecting a cure. Now I have distinctly made out that the morbid alteration, instead of being merely situated on the inner surface of the canal, usually affects the substance of its parietes; and that the mucous membrane covering it does not exhibit any sensible difference from its normal condition in other parts of the passage. Does not this circumstance alone shew the error of the accredited methods of treatment, which, it is obvious, cannot remove the morbid formation, unless by destroying the very walls of the urethra?

"2. The seat of stricture has not hitherto been accurately determined. It has usually been alleged to be most frequent in the membranous part of the urethra; whereas I have proved that it is rarely or never in this part. Hence it follows, as a necessary therapeutic consequence, that, by acting with caustic or with any cutting instrument there, the surgeon is applying his remedies on a part that is sound, and not on that which is really diseased.

"3. The same remedial means have usually been applied to all strictures alike, without reference to the part of the canal that may be affected. Now I have demonstrated that the nature of strictures differs a good deal according to their seat, whether this be at the meatus, in the spongy part, or in the sub-pubic curvature of the canal—the three most frequent seats of the contraction;—and consequently that a different line of treatment is required for each.

"4. It had been long remarked that strictures of the urethra, in which the degree of contraction was nearly the same, produced very different effects, even in cases which were apparently very analogous to each other; but the cause of such difference was never satisfactorily understood. I have shown that this depends upon the condition of the urinary bladder, which in some cases becomes atrophied and in others hypertrophied—a distinction of the very highest importance in reference to the diagnosis and treatment of the disease, since the lesions, that are apt to occur in the deep-seated part of the canal, are not and indeed cannot be alike in the two sets of cases.

"5. It is generally supposed that the chief organic alterations exist in the contracted portion of the urethra. This opinion is incorrect; for unquestionably the most serious disorders are usually seated behind the seat of stricture. It is of great importance to attend to this circumstance; to discover the real seat of a disease is the first great step to its cure; and, if the case be unmanageable, the scientific surgeon avoids tormenting his patient with the trial of various expedients, the use of which too often merely aggravates the evil.

"6. It was not only the seat, but also the real nature, of the disorders that are apt to occur in the deep-seated part of the urethra behind the point of stricture, that required to be accurately determined. This, I consider, I have done, by disclosing the long catalogue not only of chronic inflammations apt to supervene in this region of the canal and in the neck and body of the bladder, but also of the ulcerations, the abscesses, and the attendant urinary infiltrations; and again of the dilatations of the prostatic and seminiferous ducts, and of the numerous morbid states to which the prostate gland and the neck of the bladder are subject—a class of affections hitherto but ill-understood, but which certainly deserve the very greatest attention. The numerous facts, which I have collected together, must tend to throw not a little light upon this obscure department of surgical pathology, and to suggest some most useful hints for the successful treatment of many of the disorders in question.

"7. I have devoted my attention in an especial manner to pointing out the numerous mischiefs that are often produced by the use of the catheter, and of other curative means that are so frequently resorted to. You have only to cast your eyes on this large collection of pathological specimens to be struck with the great frequency of rupture of the urethra and the consequent production of false passages about the neck of the bladder, and in the part of the canal behind the seat of stricture."

M. *Civiale* is not at all friendly to the use of caustic in the treatment of strictures of the urethra. "Authors," he says, "are as little agreed among themselves as to the mode of action of the caustic, as they are with respect to the proper method of applying it. The old surgeons, as well as the English of late years, by applying the caustic from before backwards, seem not to have been aware what part they destroyed and what they spared; while the modern partisans of the plan among ourselves, by cauterising in the opposite direction from within outwards, proceed with no better chance of success. The whole subject is enveloped in a cloud of most irrational empiricism."

At a subsequent meeting of the *Academy*, M. *Mercier* called in question the accuracy of most of the preceding statements. He insisted that M. *Civiale* was entirely mistaken in his assertion that surgeons had generally supposed that the seat of stricture was in the mucous coat of the urethra, and he appealed to a paper of his own, published in the Gazette Medicale of April 1839, in which he laid down the position that "most contractions of the urethra, œsophagus, and rectum, depended upon a transformation of the substance of these canals into a fibrous tissue; and, in reference to the urethra in particular, that, as its spongy structure is only a dependence of the venous system, the process of inflammation produces the same phenomena in its cells as it does in the ordinary veins, viz. coagulation of the contained blood and obliteration of the tubes; subsequently absorption of the coagulated blood, and ultimately the entire effacement of the cells." M. *Mercier* proceeds to say that, in his judgment, M. *Civiale* is unquestionably mistaken in asserting that strictures never occur in the membranous part of the urethra—an opinion however which has been held by *Dessault, Dupuytren, Amussat,* and other eminent surgeons. He then denies the accuracy of M. *Civiale's* statement that the urinary bladder ever becomes atrophied in cases of urethral stricture: as to its tendency to hypertrophy under such circumstances, the fact has been known for centuries. Most of the other alleged discoveries, M. *Mercier* remarks, will be found in the writings of *Charles Bell* and other surgeons.

*Remarks.*—It is pleasing every now and then to hear the criticisms of one French surgeon upon another. They are in general so egregiously ignorant of what has been done out of their own "belle patrie," that it is quite a relief to us when they mutually expose each other's blunders—in reference more especially to *discoveries.*

---

### Ioduretted Injections in Hydarthrosis, &c.

M. *Bonnet,* chief surgeon of the Hôtel Dieu at Lyons, has for the last two years been trying the effect of injecting a solution of iodine in certain affections of the knee-joint. Before relating his experiments, he states that the idea of treating hydarthrosis with stimulating injections is by no means a novel one. *Boyer,* in his large work, alludes so a case where camphorated Goulard water was injected; and more recently M. *Jobert* has related three cases in which he made use of a weak spirituous solution for the same purpose. Of late years, solutions of iodine have come into much favour as injections for hydrocele and also for the fistulæ that are so frequent in the neighbourhood of diseased joints. * * * * * In puncturing a joint, the wound should always be as small as possible; and, in order that the introduction of the air may be prevented, the surgeon should first pinch up a fold of the integuments before introducing the trocar, so that the outward and inner wounds may not correspond with each other afterwards. The most convenient situation for making the puncture in the case of the knee-joint, is immediately above the patella; the limb should be fully extended at the time, in order that the articulating surfaces of the femur and tibia may be closely applied to each other. The fluid seldom flows out in a jet, as in cases of hydrocele, but only slowly and hesitatingly. It is unnecessary that the whole of it should be evacuated; indeed it is much better that it should not, and that a portion only be withdrawn. A useful precaution to prevent the entrance of the air is to keep the free end of the canula constantly pointing upwards. Solutions of iodine of very different strengths have been used as injections for hydrocele. M. *Velpeau* uses one part of the tincture and seven of water; occasionally the undiluted tincture has been employed. M. *Bonnet* used the diluted tincture in most of his cases of hydarthrosis; but then he took the precaution of discharging only a very small quantity of the articular fluid, leaving by far the greater part in the joint. Latterly he has preferred for this purpose a watery solution—1 part of iodine, 2 of the ioduret of potassium, and 8 of water—in order to avoid the possible coagulation of the articular serosity by the alcohol of the common tincture. The quantity injected should be very nearly the same as that of the fluid that has been withdrawn.

As the inevitable result of injecting a stimulating fluid into an articular cavity is an attack of acute arthritis, the surgeon must be ready to keep it within due bounds. In all my cases (of hydarthrosis), says M. *Bonnet,* the inflammation has subsided within a few days, and has never been followed by suppuration. A brief notice of these cases will be interesting.

*Case* 1.—A man, 28 years of age, was admitted into the hospital in September 1841. His health had for two years suffered much from living in an unwholesome damp situation, and also from the effects of obstinate secondary syphilis; latterly he had been affected with rheumatic pains in most of his joints. At the period of his admission, the left knee was much swollen from an obvious accumulation of fluid within the joint. Although there were no symptoms of inflammation, the pain was often excessive, especially upon any movement of the limb, so as to keep the patient from sleeping. The strength was very much reduced; but still there was no fever present. A variety of remedies was tried; but none gave any decided relief. About five weeks after his admission into the hospital, I punctured the joint, says our author, with a trocar, and gave issue to a quantity (two *centilitres*) of serosity like that of hydrocele, and immediately afterwards injected 15 grammes of tincture of iodine, which I allowed to remain in. The pain produced was considerable, and, in the course of the day, the joint became swollen and very tender; the patient was restless and feverish during the night. But in the course of 24 or 36 hours, these symptoms abated, and then the patient experienced less pain than he did before the operation. By the sixth day, he was able to rise; and five days afterwards he could walk about the ward. The right knee now began to swell and be painful; and strange to say, as the left one became well, the other filled with fluid. On the 16th of October, twelve days only after the first operation, I performed the same manœuvre on the right, as I had done on the left joint. The inflammation that supervened this time was much more severe than on the former occasion, and indeed it was alarming. Forty leeches were applied round the joint, and then fomentations and poultices; but as the tension and pain became more and more severe towards evening, I determined to puncture the capsular ligament again: about two *centilitres* of serosity (which had no traces of iodine) flowed out through the canula. From this moment, all the suffering ceased, and, in the course of two days, the joint was not larger than it had been before the operation. So rapid was the abatement of all the symptoms that, after the lapse of another week, the man was able to leave the hospital, walking without difficulty, and quite cured of his double hydarthrosis. I have seen him repeatedly since, and find that the cure has remained permanent and complete.

(Surely the second operation was a most unwarrantable proceeding: to puncture and inject a large joint for a dropsy of a few days' standing!)

*Case* 2.—A young girl had been affected with dropsy of both knee-joints for three weeks, when she entered the hospital. She seldom suffered much pain in them, and she had always been able to walk, although with inconvenience. After a few days' residence in the hospital, both joints were punctured on the same day, and about 30 *grammes* of tincture of iodine were injected into each. The inflammatory re-action was intense; during the first night, the patient was restless, feverish, and now and then somewhat delirious; both joints were much swollen, and the integuments were hot, red and shining. For three days the acute symptoms continued; they then subsided, and the swelling began to diminish. At this time I had to give up attendance at the hospital for several days, and when I resumed my duties on the 8th of June (about three weeks after the performance of the operation) I found that the patient had left the hospital nearly quite recovered.

*Case* 3.—A woman, 27 years of age, had been affected with hydarthrosis of both knees—the consequence apparently of a fall from a tree—for two years, when she came under M. *Bonnet's* care in June 1842. After using blisters for a few days, M. *Bonnet* injected into each joint some of the following solution—1 part of iodine, 2 parts of hydriodate of potash, and 15 parts of water. The inflammation that followed was very intense; and for forty-eight hours the patient could not get a wink of sleep. On the third day the pain and swelling began to abate, and by the end of another week no fluctuation could be perceived, and neither joint was much larger than in health. In the course of a month, the patient was able to leave the hospital; the swelling had almost quite disappeared, but the walking still remained difficult, although less so than before the operation.

In the *fourth* case, in addition to the hydarthrosis, which was the consequence of chronic rheumatism, there was a good deal of œdematous swelling of the integuments, and also " des fongosités dans la cavité synoviale." The complaint was of three months' standing. About 30 *grammes* of serosity were withdrawn from the joint, and as much tincture of iodine injected. The inflammatory reaction was by no means very great; not a drop of fluid oozed from the wound. As the swelling of the part did not sensibly subside, the joint was again punctured and injected, about five weeks after the first operation. This time, the inflammation excited was more severe; but the ultimate result was " une guerison presque complète.

M. *Bonnet*, after describing another case, goes on to state that in no instance in his practice has the iodine injection caused suppuration of the joint or any other severe accident; the inflammatory action, although sometimes very considerable, almost always subsided in two or three days. There is every reason therefore to believe that many cases of simple hydarthrosis may be most successfully treated in the manner now described.—*Bulletin de Therapeutique.*

---

### On the Use of the Hydriodate of Potash in Rheumatism.

Dr. *Aubrun* tell us that, for some years past, he has been in the habit of using the hydriodate in acute as well as in chronic rheumatism with marked success. The doses that he often exhibits are large—from one to four scruples and upwards in the course of the twenty-four hours. He suggests that the probable cause of the salt disagreeing with certain people, even when administered in small quantities, is the admixture of free iodine with it—a substance which the stomach can seldom bear for any length of time with impunity. The only inconveniences which Dr. A. has observed to attend the use of the hydriodate are, a more or less copious salivation, a bitter taste in the mouth, and redness and a sense of heat in the throat. According to his experience, it is decidedly more efficacious against acute than against chronic rheumatism. He very generally premises the use of bloodletting and other antiphlogistic measures, and then exhibits the salt in solution along with some syrup of poppies. But if the constitution be feeble, recourse should be had at once to the hydriodate. Dr. A. remarks that patients, treated with this medicine, are less subject to stiffness or rigidity of the muscles and to swellings of the affected joints than when the cure of the disease has been obtained by the use of other remedies. He has never known any disagreeable effects to follow its administration, even when this was continued in full doses for a considerable time: no wasting of the testicles in the male, or of the mammæ in the female subject. He concludes, after relating several cases of severe acute rheumatism successfully treated, by saying that the action of the hydriodate is altogether depressing (hypostenisant) and resolvent; and that its remedial powers seem to be the greatest when the constitution of the patient is weak, or after one or more bloodlettings, if the patient be robust and plethoric.—*Gazette Medicale.*

---

### ON THE TREATMENT OF OPHTHALMIA WITH CAUSTICS.

M. *Bernard* of Paris has recently published a work, entitled, "the Ectrotic or abortive Method applied to the Treatment of Ophthalmia in general, and of Purulent Ophthalmia in particular." We extract from the Belgian Journal, Annales d'Oculistique, the following notice of its contents.

. . . . . " When M. *Sanson* first employed cauterisation and excision in the treatment of blenorrhagic ophthalmia, the practice, being at variance with all the established usages of the schools, was deemed rash and improper. The means, that were hitherto almost invariably used, were free and repeated blood-letting, nauseants, calomel, &c.; and although they often failed in saving the eyes from total destruction, surgeons seemed to be but little disposed to make any change in their mode of treatment. To M. *Gouzée*, principal physician of the Military Hospital at Antwerp, the profession is not a little indebted for having most convincingly demonstrated the admirable efficacy of cauterization in numerous cases of purulent ophthalmia. His great experience has led him to place little or no reliance on bloodletting for the cure of this formidable disease; and, indeed, in not a few cases, it is in his opinion of positive mischief. Let it not, however, be supposed, that he altogether proscribes the use of this and of other antiphlogistic and soothing means: he admits their advantage as accessory remedies, but rejects them as the sole or principal means of cure. The nitrate of silver, applied at an early period of the disease, over the entire extent of the mucous surface of the eyelids, is *the* remedy, " par excellence," against gonorrhœal ophthalmia. Its employment, directed by a prudent and experienced hand, is never followed by any inconvenience. The experience of M. *Ricord*, of Paris, fully confirms the accuracy of his Belgian *confrere*'s statements. It is not of very much consequence whether the nitrate be applied in a solid or in a liquid form; each method has its advantages in particular cases. The great object of the surgeon should be to have recourse to the remedy before any sensible change has taken place in the texture of the cornea: if the disorganising process has once fairly commenced, we can have but little hope of saving the eye.

*Remark.*—Is it rather droll that the name of *Guthrie* should not even be mentioned by our *confrere* on the other side of the Channel, in reference to what he calls the " ectrotic treatment " of purulent ophthalmia. We had always foolishly believed that our countryman had had something to do with the introduction of the practice in question.—(*Rev.*)

---

### ON DISEASES OF THE MAMMA.

According to the inquiries of M. *Tanchon*, the frequency of mammary tumors is decidedly on the increase in France. In 1830, he says, 668 persons died of cancer in the department of the Seine, and in 1841 not fewer than 889. Of these numbers, 595 deaths occurred in Paris alone during the first, and 779 during the latter of these years.

M. *Tanchon* proposes various means to check the development of diseases of the mamma. Among other remedies, he recommends the employment of gentle compression with bags containing the following powder —

Ioduret of potassium, 5 parts, powdered sponge, 10 parts, muriate of ammonia, 40 parts, and muriate of soda, 10 parts.

There is another discutient powder—composed of 20 parts of powdered sponge, 1 part of nitrate of potash, and 1 part of orris powder—which he occasionally uses. He is strenuously opposed to the excision of the mamma, and to the application of any caustic or irritating substances to it.

---

### Etiology of Deformities; the Operations of Myotomy and Tenotomy; the Claims of M. Guerin, &c.

A short time ago, there was an unusually prolonged discussion on these subjects at the Royal Academy of Medicine.

As the discussion had been introduced by the two leading orthopœdists of Paris, MM. *Guerin* and *Bouvier*—both of whom have hygienic establishments or 'Maisons de Santé' in the environs—and seemed to be rather a dispute about some rival claims of these gentlemen than a theme for scientific remarks, the leading members were unwilling to take any part in it.

M. *Guerin*, however, having put forward some most extraordinary pretensions to certain alleged discoveries touching the pathological history as well as the remedial management of deformities of the limbs and other parts, MM. *Velpeau* and *Gerdy* thought it necessary to expose the egregious errors of that gentleman, errors which seemed to arise more from profound ignorance of what had been already done and published, than from wilful plagiarism or dishonesty. It may be interesting to the English reader to be made acquainted with the leading topics of a controversy that was carried on, for several meeeings, with more than usual animation, if not acrimony, especially as some of them are objects of considerable interest to the physiologist and surgeon.

One of the grand discoveries of M. *Guerin*, on which he descants upon all occasions, is that almost every variety of deformity of the limbs and trunk is owing to morbid muscular contraction, that has taken place either during intra-uterine life or after birth. This doctrine is quite true of many, but certainly not of all, cases. M. *Gerdy* very ably proved that the causes of deformities are various: he reduced them under four heads:—1, irregularities in the primitive formation of the bones; 2, alterations ulterior to the formation of the bones; 3, fibrous or fibro-cellular indurations; and 4, muscular retraction. He remarked that M. *Guerin*, having devoted himself almost exclusively to one class only of diseases, is but ill qualified to take those large and comprehensive views of morbid action, which they only, who embrace the entire domain of disease in their practice, can duly comprehend "A medical man, who gives himself up entirely to the practice of any speciality, cannot take in the entire horizon of science, or easily appreciate the numerous and varied causes of osseous irregularities."

It was not, however, to the doctrine itself—viz., that the most frequent cause of deformities, especially of such as are congenital, is a tonic contraction of the muscles—as to the claim of M. *Guerin* to its discovery, that the chief opposition was made. M. *Velpeau* most convincingly shewed, by quotations from many well-known writers during the last 100 years, that the truth of this pathological dogma has been abundantly well known for a great length of time. One or two of these will suffice for our purpose.

*B. Bell*, in the sixth vol. of his System of Surgery, thus expresses himself: "The limbs are apt to be twisted in a variety of ways. In some cases, the bones are more or less diseased, and in others, the muscles are in a state of contraction; while, in a third set, both the bones and the muscles are affected."

.. .. .. .. .. "The most frequent cause of the irregular conformation of the limbs is the contraction of the flexor muscles of the leg and fore-arm;" and in a subsequent passage he says, "since the deformity of the limbs is the effect either of the contraction of the muscles attached to them, or of curvature of the bones, it is obvious that we must vary our remedies according to the nature of each case."

In a most interesting passage in his great work, M. *Beclard* thus announces his views as to the etiology of many congenital malformations. "I conclude," says he, "from all the observations which I have previously recorded, and the reflections to which they have given rise, that acephalous monsters have experienced, at an early period of intra-uterine life, an accidental disease which has caused the atrophy or the destruction of the medulla oblongata and upper part of the spinal marrow, and that all the irregularities which they exhibit are the natural and the more or less direct consequences of such a lesion."

But there is no author whose observations are altogether so lucid and conclusive on the subject which we are now considering as the late M. *Delpech*, the great ornament of the Montpelier school. In his Treatise on Orthomorphy—a work, says M. *Bonnet*, replete with most valuable ideas, which the researches of subsequent writers have only confirmed and developed—after relating a case of club-foot that had followed a disease of the internal popliteal nerve, remarks:— "This case shews that when a nerve, or any of its principal branches, happens to be exposed to an irritative action, it may then transmit the irritation to all the muscles under its influence, so that they deliver themselves up to a permanent effort of shortening, that is apt to induce a most striking change in the form, by altering the relation and mutual adaptation, of the bones." .. .. .. ..

"What happens to a limb, may equally occur in the trunk under similar circumstances; and not only diseases of the nerves themselves, but those also of the medullary pulp from which they arise, may exert the same influence on the muscles of the trunk, as on those of the limbs to which they are sent."

So much for the doctrine of muscular contraction being the cause of deformities, and for the claims of M. *Guerin* to any important discovery on the subject. We now proceed to make a few remarks on the "Character of Subcutaneous Wounds," a subject intimately connected with the treatment that of late years has been so much in vogue in all cases of distortion of the limbs, &c.

*John Hunter* was the first who very clearly and emphatically pointed out the marked difference in the results of lesions, according as the integuments are, or are not, injured at the same time. As long as the affected parts are protected by their natural coverings from the external air, there is usually but little tendency to severe inflammatory action: when they are exposed to the air, the process of suppuration very generally is established. M. *Estor*, the clever translator of *Johh Bell's* work on Wounds, interpreting the English doctrine as to the phenomena which take place in a wound that is not exposed to the air, remarks; "The divided parts re-unite without being attended by any of the usual symptoms of inflammation, in virtue of a property similar to that which presides over the act of nutrition. This holds true in an especial manner if the injured tissues remain covered with their natural integuments, such injuries healing very generally without the supervention of any inflammatory disturbances."

Nowhere have the Hunterian doctrines been so well understood as in the school of Montpelier, and we are, therefore, not surprised to find that it was *Delpech*, a fervent admirer of the great English genius, who was the first to propose and perform the operation of dividing the tendo-Achillis by introducing a narrow bistoury under the skin, and leaving the divided tendon protected from exposure to the air. It was he who first gave practical effect to the important principle that had been propounded by *Hunter*—the principle that injuries of any part are rarely followed by much inflammatory action, when the skin over it remains unbroken. Indeed, we find that *Hunter* himself, on one occasion, divided the tendo-Achillis in a dog with a cataract needle, and subsequently that *Bell* recommended, in certain cases of contracted fingers, the section of the lateral digital ligaments by slipping a narrow bistoury under the skin.

*Brodie* also, more than thirty years ago, applied the same principle to the treatment of varicose veins: and, lastly, *Delpech* distinctly announced the important proposition that the section of tendons is unaccompanied with any danger, if it be performed by simply puncturing the skin. Since his time, *Stromeyer* of Hanover, *Dieffenbach*, and MM. *Guerin*, *Duval* and *Bouvier* have done much to bring the operation of tenotomy into repute. M. *Guerin* has certainly carried it to a very extravagant length, and there are really some grounds for the fear of M. *Velpeau*, that the operation may come ' s'epancher a pleins bords dans la science et dans la pratique.' The success of tenotomy will depend much upon the particular tendon that requires to be divided. If, as in the case of the tendo-Achillis, the part be simply invested with cellular tissue, the operation will most probably be quite successful; but if, as in the case of a digital tendon, it be surrounded with a fibro-synovial sheath, the adhesive process between the two divided ends is almost always imperfect, and the movements of the member are invariably more or less completely lost. M. *Bonnet* alludes to this difference in the following passage: "The cures effected by tenotomy in club-foot, wry-neck, &c. made many surgeons hope that the same success might attend the operation in contractions of the hands and fingers. Experience, however, has not confirmed these expectations; for we find that, in all the cases hitherto made public, the fingers may have been straightened, but their power of flexion has been lost."

M. *Doubovitski*, who himself submitted to this operation, has made some judicious reflections on this subject. He says: "Tenotomy has been applied to deformities of the upper extremity with the same hopes as it was to those of the lower one; and yet there is a material difference in the two cases. In the case of the latter, form or shape is almost everything; by rectifying this, we are certain that the patient may, more or less completely, recover the use of the member; but in the former, the shape is only a secondary object—the power of using the member is the main one. The hand is an organ of prehension, the foot one one of mere support: there lies the difference. For this reason there will be always greater success in tenotomy applied to the former than to the latter of these parts."

M. *Guerin*, however, has boldly asserted that the flexor tendons of the fingers may be divided either at the wrist, in the hand, or in the phalanges, and yet regain their normal movements. But this, like most of the other announcements of this gentleman, must be received with a good deal of reservation.—*Annales de la Chirurgie.*

---

### M. Royer-Collard on Hygiene.

The title of the Memoir read at one of the recent sittings of the Academy of Medicine, from which the following extracts are made, is "Hygienic Organoplasty, or Comparative Hygiene, on the means of artificially modifying the forms of living things by regimen."

The scope of scientific hygiene is not merely to preserve health and prevent the development of disease; it aims also at ameliorating and perfecting the various instruments of life, and at promoting the full development of all the powers of the system. By means of judicious management, we can either moderate or excite the vital powers, augment or diminish their energy, and modify in a variety of ways the form, the size, and the activity of the several parts of living bodies. We all know how much has been done in this respect as regards plants and many of the lower animals: may not the human frame, although more curiously and wonderfully formed, be susceptible of somewhat similar changes by a due education of all its powers and faculties?

### *Regimen.*

"Under this term we include not merely the diet, but also the regulation of dress, of exposure to atmospheric changes, of the exercise of the moral and mental powers, and, lastly, of the functions of generation. As every part of the body, solid as well as fluid, is continually undergoing the processes of destruction and reparation, it is quite obvious that the substance or tissue of the different organs must materially depend upon the nature of the food that is taken into the stomach, and the powers of the system to assimilate it. Then, consider how much we are all influenced by the conditions of the weather, by the heat or cold of the atmosphere, its dryness or moisture, by the state of its electricity, &c.! The influence of exercise is not less conspicuous than either of these; a due degree of it quickens all the powers of nutrition, promoting the development of every part, animating all their functions, and causing the muscular system more especially to be developed with unusual vigour. Then again, that the *breed*, so to speak, of any tribe of animals is not a little modified by certain conditions in respect of the process of generation, will be admitted by every one who has examined the question. The character of the offspring is as much influenced by the state of the parents' health, as that of the individual is by the other circumstances we have just been mentioning. And lastly, as to the moral influences, their nature is indeed different, but their operation on the health is not less indisputable."

*Influence of Food on Plants and Insects.*

"Anatomy and physiology have been indebted for the chief progress, which they have made during the present century, to the comparative study of man with the lower animals. Pathology has scarcely yet entered upon this field of enquiry; and hygiene is still further in arrear; although it, perhaps more than any other department of medical study, might derive the most important advantages from this pursuit. The practice of agriculture, the rearing of cattle, and the education of domestic animals, have amassed a great treasure of the most interesting and instructive facts. But it is chiefly in the vegetable kingdom that the influence of cultivation is most conspicuous. What an innumerable number of varieties have often been obtained from a single species! Inert or even poisonous plants have been transformed into vehicles of nutritious food; small insignificant flowers into gorgeous heads of blossom; the sexual parts are converted into petals, leaves into fruit-buds, and roots into branches. M. *Liebig* tell us that the fineness of the Italian bonnet straw altogether depends upon cultivation, and adds that, "if the plant be supplied with carbonic acid and other matters which it requires, azote excepted, it will produce leaves but no grain, and sugar and fecula but no gluten." Modifications, scarcely less wonderful, have been observed to occur in the animal kingdom. M. *Dumeril* has communicated to me some interesting facts respecting the changes which different kinds of food produce in the size and form of some insects. For example, the sexual character in bees seems to depend in a great measure on their mode of living and on the food which is supplied to them. Among the larvæ destined to become the females, only some of them acquire the attributes of their sex; the others remain neuter. The former are lodged in cells that are larger, thicker, and very different from the rest; and thither the working bees bring a pulpy nutritious food, the colour and savour of which are quite peculiar. It is chiefly this alimentation that causes the development of the generative organs in the queen or breeding bees. At the side of the cells occupied by them, are other cells occupied by other larvæ. Now these, without becoming precisely females, profit by their position; for they are generally larger than the strictly neuter bees, and moreover they afterwards produce a certain number of ova, the larvæ from which become the male insects. If by accident any of the female bees perish in the comb, the working bees forthwith set themselves to repair the loss; they enlarge the cells of two or three of the larvæ, and commence to bring them a supply of the *royal* nourishment: in this manner new females are provided for the supply of the hive. The knowledge of these circumstances has led physiologists to some most interesting discoveries. It has been found that we can at will change the female into neuter, and the neuter into female larvæ. Similar phenomena have been observed in ants."

*Development arrested or Modified.*

"In the higher classes of animals, our attention is drawn to the singular transformations which have been effected by artificial means in different animals while in the fœtal state. M. *Edwards* has succeeded in preventing tadpoles advancing to their complete development and becoming frogs, by depriving them of air and light; the animals continue to grow in size and strength, but still retain their fœtal form. In the case too of the eggs of the common fowl that are artificially hatched, it has been found that, if the heat be applied unequally, monstrosities (which may be 'calculées d'avance') are the result: in one case big extremities with a minute head, and in another a small body with a very big head, may be induced. As we approach the human species, the changes that may be effected by domestication acquire additional interest. Need we do more than merely allude to the very wonderful difference in different races of dogs and horses?—all the result of breeding and education. Not less remarkable are the changes that have been made in the breeds of sheep and large cattle, by the labours of the farmer. The principles, on which all such changes have been effected, are on the one hand the judicious selection of the animals for breeding, and on the other, of the food with which they are provided. To such perfection are these matters understood in the present day, that the experienced farmer can at once tell you what sort of food the animal must be chiefly fed upon, according as the object may be to fatten it, or to increase the plumpness of the muscles, to promote the flow of the milk, or increase the growth of the wool or hair. The system has been applied even to some kinds of fish; for it has been found that, if they be castrated and then kept in damp soft moss, they will often acquire a most unusual size."

*The Hygiene of Children.*

"When we have studied successfully all the orders of the zoological scale, in the mechanism of their functions, and when we find that in each individual without exception there is constantly present the same physiological phenomenon, we may then with almost perfect confidence predict, that this phenomenon will be observed in man also—who, although peculiar in his form and higher organisation, resembles other creatures in his general nature and in the predominant characters of his system. We are thus led to draw some important hygienic instructions, from what we observe to take place in the case of the lower animals. How much of the future healthfulness of life depends upon the appropriate management, especially in respect of food, of the infant during the first twelve months! Most of the cases of rickets, and of deformities from other causes, are directly traceable to imperfect or improper nutrition during this early period."

*The Effects of Training.*

"The effect of the *training*, to which pugilists, jockeys, and others submit in order to bring themselves into what is called *condition*, is truly remarkable. All the subcutaneous fat becomes quickly absorbed; the cellular tissue becomes firm and unyielding, so that any blood that is extravasated under the skin is circumscribed to a small space; the skin becomes smooth and clear; the muscles are unusually firm and prominent; the belly small; the chest full and well expanded; the general sensibility of the body very materially diminished; and the spirits are buoyant and elastic. It might be supposed that men, who had frequently submitted to the regime of training, would suffer for such effects in their health afterwards; but it would seem not; for many of the leading English boxers have lived to a green old age, and retained much of their early vigour to the last.

Jockeys are certainly more unfortunate; but then with them, the great object is simply to diminish their weight, without any regard being paid to their muscular strength at the same time.

"In the case too of divers, we find that much of their skill depends upon the regimen to which they submit themselves. *Spalding* remarked, in his own case, that he always consumed the air in the diving-bell more rapidly, when he lived much on animal food, and drank malt or spirituous liquors. Whenever he wished to remain an unusually long time under water, he eat nothing but vegetable food, and confined himself to water only for his beverage. Divers, like runners, use various means to develop their respiratory energies, by the regulation of their diet and exercise; and some of them have attained truly wonderful power in this respect."

After some explanatory remarks on the nature of *training*, as resorted to by the various *athletæ* to whom we have alluded, our author makes the following reflections:—

"Nothing is more simple, and withal nothing is more physiologically skilful at the same time, than the regimen followed by these men. It is exactly the application of the famous Cyclic rule of the Methodists, related by Cœlius Aurelianus: 'recorporativis utendum viribus, ita ut, rejectis vitiosis carnibus ac renascentibus novis, reformata organa redeant ad sanitatem.' By purging away all offensive lodgements from the body, by bringing the skin into a soft smooth state by sweating, and by then supplying the system with plenty of wholesome nutritious food, as well as by the regular use of moderate exercise, can we at all wonder that the body should acquire greater energy and power of endurance? It is only surprising that medical men should always be so far behind in reaching the natural and right way, and be obliged to learn from men of no education, and who have derived their skill only from repeated observation of a few simple facts. From the preceding statements it is not difficult to perceive that some valuable therapeutic suggestions may be derived. Many of the various forms or degrees of health may be successfully modified by a systematic regimen, carried out for a certain length of time with judgment and perseverance; and there are not a few morbid states of the constitution that might be more benefited by such a simple means, than by all the elaborate prescriptions of the most wise and learned."—*Gazette Medicale.*

---

### M. DUMAS ON THE MUTUAL HARMONIES OF VEGETABLE AND ANIMAL LIFE.

For a length of time chemists have been in the habit of recognizing three neutral azotised constituents in animal bodies, albumen, fibrine, and caseine. About a twelvemonth ago, M. *Boussingault* and myself endeavoured to prove that these three component principles exist in plants also, and that therefore they pass already formed into the bodies of herbivorous animals, and thence come to enter into those of the carnivorous. According to the views which we then explained, it appeared to us that to plants only belongs the privilege of forming these products, which become afterwards assimilated with the bodies of animals. We have extended these principles to the formation even of fatty matters, which in our opinion, have their origin solely in the vegetable world, and which subsequently play the part of a combustible in animal bodies. We pointed out the necessity of grouping together all the substances of organic chemistry which possess the property of passing into the state of lactic acid by fermentation, and which, like sugar and the fecu-lent grains, form so important a part of the food of man and the lower animals: they are all really and truly the products of plants alone, developed by the force of vegetable life. The following table exhibits our views on this subject:—

| *The Vegetable* | *The Animal* |
|---|---|
| Produces neutral azotised matters. | Consumes neutral azotised matters. |
| ——— fatty matters. | ——— fatty matters. |
| ——— sugar, fecula, gum. | ——— sugar, fecula, gum. |
| Decomposes carbonic acid. | Produces carbonic acid. |
| ——— water. | ——— water. |
| ——— the ammoniacal salts. | ——— the ammoniacal salts. |
| Disengages oxygen. | ——— oxygen.* |
| Absorbs caloric. | Produces caloric. |
| Absorbs electricity. | ——— electricity. |
| Is an apparatus of reduction. | Is an apparatus of oxydation. |
| Is motionless. | Is locomotive. |

A graminivorous bird finds in the corn that it lives upon all the elements necessary for its nutrition. A dog finds in bread whatever its organisation requires for its sustenance and growth. A cow finds in grass all the elements that serve not only for its own nourishment, but also for the formation of milk that is so rich in caseine. It thus appears that cereal grains contain, in addition to their saccharine and amylaceous elements, the azotised materials which are found in all animal bodies.

" From what has been now said, we may draw the following two fundamental principles respecting the feeding of animals.

1. That neutral azotised materials constitute an indispensable element in the food of animals.

2. On the contrary, that animals can, to a certain point, do without fatty matters, and wholly without feculent and saccharine matters; but on this condition only, that the fatty substances are replaced by proportional quantities of fecula and sugar, and *vice versâ*. The privation of fatty matter does not for a time compromise the life of the animal; nevertheless it exercises a peculiar effect which deserves a few words' notice. The necessity, which all animals feel to have food that contains the neutral azotised matters which exist in their own organisation, shews pretty clearly that they cannot create these substances for themselves. But to place this fact in a more distinct point of view, we have only to follow these azotised matters after being received into the stomach, and to find out what is their final destination. Now it is not difficult to prove, that they are essentially represented by the urea—which in man and in the herbivora constitutes the leading element of the urinary secretion—and by the uric acid, which in the case of birds and reptiles plays the same part as the urea. Allowance being made for the excrementitious matter from the bowels, we may assume that an adult man absorbs every day an amount of neutral azotised matter sufficient to represent 15 or 16 *grammes* of azote—a quantity of azote found in about 30 or 32 *grammes* of urea. May we not very reasonably conclude that the azotised ingredients of our food serve to produce this urea, and that every effort of the animal system is directed either to assimilate it to itself, when it has occasion for it, or to convert it into urea? The truth of this opinion becomes doubly sure when we remember that the study of the phenomena of respiration shews us that the fatty matters disappear from the animal organism in consequence of a veritable combustion, that the amylaceous and saccharine matters are also consumed (brulés), and lastly, that the difference between urea and the neutral animal matters from which it is derived, is most exactly represented by a phenomenon of combustion."

---

* Is there not a mistake here? The living animal surely *consumes* rather than *produces* oxygen. There is probably a typographical error, as the repetition of the word " produces" in the next line seems to imply that its converse had been used before.—*Rev.*

. . . . . . . . . . . . . . " It clearly follows that the quantity of azote, which our food contains, gives us a clue to calculate the powers of assimilation and the quantity of aliment required for the due sustenance of life; the azotised matter being the substance that is essentially assimilable, and that which constitutes the web and woof, so to speak, of the whole system. As this element (the azote) is found almost entirely in the veins, under the form of urea, it remains for us to enquire what is the nature of the urea, and in what respect it differs from the neutral azotised matter whence it is derived. The beautiful researches of M. *Vohler* has shewn us that the urea may be generated by a modification of the cyanuret of ammonia—itself formed by an oxyde of cyanogen and an oxyde of ammonium. There are thus given off from the animal body four different oxydes—carbonic acid, cyanic acid, the oxyde of ammonium; the two last combined and modified produce urea."

. . . . . . . . . . . . " If we reflect on the circumstance that the blood constitutes a solution of the solid materials of the economy, we can readily understand how it is so important that the process of digestion should be incessantly restoring to the blood its constituent elements, in order that these elements, which are continually undergoing the act of combustion, should not be taken up again by the impoverished blood, and conveyed to the different organs which contain them. And to apply these principles to the azotised matters of which we have been treating, we should say that, if it is indispensable that the alimentation of a man should furnish a daily supply of from 100 to 120 grammes of dry azotised matters, it is because we know that nothing can prevent the system from losing in that period of time such a quantity of these matters by respiration and the combustion that is the consequence of it."

. . . . . . . . . . " In concluding his remarks M. *Dumas* said :—" The Academy will be able to judge from the preceding observations what is the nature of the researches which I have been engaged in, with the view of establishing an exact balance between the quantity of fatty albuminous and saccharine matters consumed, and the amount of caloric produced by their combustion in the body of living animals. It will also see, and we hope with feelings of some interest, what experiments we have devoted our attention to, for the purpose of establishing on a certain basis the rules to be followed in calculating the expenses of maintaining our soldiers, work-people, paupers, and prisoners. These are matters of the highest importance in an economical as well as in a medical point of view, and deserve attention from the political philosopher no less than from the physiological enquirer."

---

### A Treatise on Pathological Chemistry.

Such is the title of a work, recently published in Paris by Dr. *Lheritier*, on the Chemical Constitution of the Solids and Fluids of the Human Body, in a physiological and pathological point of view. We have not seen the work ourselves, and are indebted for our knowledge of its contents to a review of it in one of the French journals, from which we have made the following extracts :—

" Chemical analysis is now more than ever appealed to by medical men, to lift up the veil that covers so many of the mysterious wonders of animated nature from our knowledge. Already it has done much, and promises much more to the diligent enquirer. What the science of acoustics has, with the aid of the stethoscope, done in aiding our diagnosis of thoracic diseases, chemistry will do in explaining the leading phenomena of many other diseases. We have chemical lesions, and we have physical lesions; a medical chemistry as well as a medical physics. That chemical analysis, like acoustic, microscopic, or any kind of analysis, will lead to some valuable discoveries in physiology and pathology, few will in the present day be inclined to dispute. The great object must be to direct its course aright."

M. *Lheritier* first examines the chemical characters of the three animal fluids —the chyle, the lymph, and the blood—which are most immediately concerned in the phenomena of nutrition. His experiments on the chyle lead to the conclusion that this fluid is not more highly animalised in carnivorous than it is in herbivorous animals; the quantity of azote being nearly the same in both cases. The composition of the lymph is so similar to that of the chyle, that they are scarcely to be distinguished.

M. *Lheritier* is a firm believer in *Harvey's* and *Hunter's* doctrine of the vitality of the blood. In his opinion, it may be regarded as the centre and source of life with much more propriety than the nervous system; for the solids die instantaneously when deprived of blood, while some of them will continue to act even for hours after the destruction of the brain and spinal marrow. The numerous experiments, which he has performed on the coagulation of the blood, have led him to attribute this change entirely to a chemical agency, which does not commence until the blood has lost its vitality.*

As to the composition of the circulating fluid, our author says that, besides a certain amount of combined carbonic acid, it contains also a small portion of the free acid. The never-ceasing generation of this substance by the combination of the oxygen in the respired air with the elements contained in the blood is, in his opinion, the cause of animal heat.

M. *Lheritier* confirms the accuracy of many of MM. *Andral* and *Gavarret's* statements respecting the changes that the blood undergoes in acute diseases, and dwells especially upon one result of their experiments, viz. that the proportion of the red globules becomes decidedly diminished, when an acute disease has lasted for some time. This fact explains some phenomena that are otherwise not very intelligible. Enfeebled and weakly persons are more liable to inflammatory attacks than those who are more robust and plethoric, in consequence of the proportion of the fibrine to that of the red globules in their blood being relatively, although not actually, greater than in health.

The circumstance of the blood from a punctured vein, as in ordinary phlebotomy, becoming of a bright red colour after a certain amount has been drawn, has more than once announced to Dr. L. that it was high time to stop the hæmorrhage, unless syncope was wished for. He attributes this phenomenon to the impaired activity of the nervous system interfering with the accustomed change of the arterial into proper venous blood.

The blood of diabetic patients is found to contain saccharine matter, especially if the blood has been drawn soon after a meal; the coagulum is usually very soft, the proportion both of fibrine and red globules being below the normal standard.† In chlorosis the proportion of the red globules first, and subsequently, if the disease lasts long, of the fibrine also, becomes very considerably diminished: when

---

* Dr. L., in a subsequent part of his work, suggests the practical precept, that *the more quickly that the process of coagulation is found to take place, the less blood should be drawn,* in any case. It is necessary to distinguish between the mere rapidity and the completeness of coagulation. For example, in adynamic fevers the blood coagulates more quickly than in inflammations; but the coagulation, as every one knows, is much less perfect. The slowness of the act of coagulation and an increase in the absolute or relative proportion of the fibrine to the other constituents of the blood, are the main causes of the formation of the buffy coat.

† Dr. *Lheritier* mentions that he has seen decidedly good effects from powdered colomba root and the ioduret of iron, in conjunction with an animal diet, in several cases of diabetes mellitus.

the latter change takes place, there is very generally greater or less œdema of the lower extremities. Dr. L. has some peculiar notions on the state of the blood in genuine scurvy; he does not admit that this disease is the result of an alkaline condition of the fluids. He seems to have little faith in the curative effects of lemon and other vegetable juices, and suggests, in lieu of them, nitrate of potash and muriate of soda!—this almost reminds one of the proverbial advice to the drunkard to "take a hair of the dog that bit you,"—which he says are tonics and have a marked influence in reddening the blood.

Some of the remarks of Dr. L. on the changes of the urine in different diseases are interesting. He made many experiments on the albuminous state of this secretion, which, some years ago, was supposed to be pathognomonic of the *morbus Brightii.* It is now generally acknowledged that, in other diseases, especially in those of the heart and of the liver, the phenomenon is of not unfrequent occurrence. Whenever it is present, we have some reason to suspect that the state of the blood itself is more or less altered from the standard of health.—*Journal des Connoiss. Medico-Chirurg.*

---

### MEMORANDA ON SUPPURATION.

The phenomena of the process of suppuration have been lately attracting much notice among many of the French pathologists. Two of the ablest enquirers are M. *Darcet* and M. *Conté,* both of whom have published very interesting memoirs on the subject. The former of these gentlemen has pointed out with great accuracy the changes which purulent matter undergoes on exposure to atmospheric air, and has deduced from his observations on this subject some ingenious conclusions touching the development of what have been called consecutive or metastatic abscesses.

If pus, he says, be exposed to oxygen, it soon separates into two parts—one nearly solid and membraniform, the other a sanious liquid of a dark colour and which gives off carbonic acid. If the action of the gas be continued, this liquid quickly putrifies, and emits a strong ammoniacal odour; the membranous portion remains nearly unchanged. When the former is injected into the veins of an animal, all the symptoms of an adynamic purulent infection of the system speedily make their appearance: the whole of the circulating mass becomes more or less completely vitiated. The poisonous action, which goes on "de proche à proche," is compared by M. *Darcet* to that of a leaven in a fermentable fluid.

When purulent deposits are absorbed, without giving rise to any constitutional disturbance, our author is of opinion that the matter is carried off by the kidneys; for the urine in such cases will be usually found to be more or less charged with albumen, as M. *Martin Solon* has observed to be the case at the period of the pustular desiccation in small-pox. Now, as there is albumen in the liquid portion of purulent matter, we may naturally suppose that this element has passed with the serosity into the urinary secretion. The semi-solid membraniform portion remains, he thinks, behind in the seat of the abscess, and may afterwards be recognised, at least in some cases, as a yellow amorphous adipocire-like substance, such as *Dupuytren* and some other pathologists have observed in various organs, and especially in the brain. M. *Darcet* has several times inclosed some purulent matter in a permeable bag, and then placed this among an absorbent powder; the fluid portion of the pus soon disappeared, and nothing was left behind but the solid membranous portion of which we have been speaking.

M. *Conté,* in his essay, has entered at some length into the history of *pyology.* It is often very useful in medical literature to look back upon what our distinguished predecessors have done; for really they seem to have been more assiduous, and certainly more unprejudiced, observers of the phenomena of nature than their more boastful children. Let us follow M. *Conté* in a few of his remarks.

*Pringle,* he says, has observed that the serosity of the blood, if exposed for some time to the temperature of the animal body, becomes turbid long before there is any appearance of putrescency, and deposits a white sediment not unlike to pus: he therefore concluded that it (pus) is derived from the watery portion of the blood. *Van Swieten* remarked that purulent matter exudes in a serous form; and *Nicholas Romagne,* in his thesis published at Edinburgh, distinctly maintained that it is from the serum of the blood alone that pus is formed. We know also that *John Hunter* and Sir *E. Home*—although their opinion was that pus is a secretion from the entire blood, and not merely from one part of it—have stated that, if we carefully prevent the pus from remaining on the surface of an ulcer by continually wiping it off, nothing but a transparent limpid serum is then secreted.

Would it be unreasonable to conjecture that purulent matter becomes acid in a few moments after being secreted, and that thereby its albumen becomes precipitated? This idea may appear very fanciful to many surgeons, who are but little conversant with the nature and results of chemical action; but let them remember that it may be with pus as it is with urine, which every one knows is quite transparent when voided, but may become thick and muddy almost immediately afterwards.

It is very possible that electricity may have something to do with the change. *Majendie* remarks, in his Treatise on Physiology, that "the action of the galvanic pile coagulates serum, and causes the development in it of globules which are very analogous with those of the blood."

We know that an abscess contains at first nothing but serum; subsequently there is a thin puriform fluid, like skimmed milk, and at length a thick and opaque pus (*Burdach.*) The same succession of phenomena is observed in the successive changes of the contents of a variolous pustule. May we not recognise in such facts as these a process altogether similar to that observed by *Hunter* and *Home* in ulcers, and also on the surface of mucous and serous membranes?

Let us now see whether chemistry affords any confirmation of these views.

In M. *Andral's* work on Pathological Anatomy, we meet with the following passage:—"Pus has been analysed by many chemists. *Schwilgué* found that it was composed of albumen in a peculiar condition, of extractive, of a fatty matter, and of various saline substances. According to this analysis, pus would seem to differ from the serum of the blood only by the peculiar state in which the albumen exists, and also by the presence of extractive matter. The nature of this last substance has not been well ascertained: some regarding it as an animal matter, not analogous to anything existing in the healthy body; while others view it as a mixture of albumen and fibrine, or as fibrine which has become spontaneously incoagulable and inorganisable. According to *Pearson,* it is an animal oxyde; according to others, it is of a cheesy character."

M. *Gendrin,* who has written so largely and elaborately on inflammation and its consequences, writes thus on the question under consideration:—"It seems to result from all our observations that laudable pus is composed of soluble albumen in small quantity, and of albumen united with fibrine: it is the union of these two substances that constitutes the pulverulent matter which is precipitated in water."

M. *Donné* is of opinion that the precipitate consists of albumen coagulated by muriatic acid—which, according to him, is always generated during inflammatory action.

While all authors are agreed as to the presence of albumen in pus, there is no little difference of opinion as to the existence or non-existence of fibrine in it. Many writers distinctly deny that it is present at all; and even those who believe otherwise are forced to admit that it exists in a peculiarly modified condition, which gives it the appearance and somewhat of the character of cheesy matter. Does not this very circumstance of the fibrine—if indeed there be any at all present—being in an essentially altered state, confirm in some degree the idea that pus is derived mainly or entirely from the serum of the blood?

Supposing that the truth of this be admitted, the question now presents itself —in what manner does the serum become transformed into purulent matter?

Dr. *Conté* suggests the following explanation:

"It is admitted that, during the process of inflammation, the course of the globules of blood becomes slackened at first, and is afterwards completely arrested, so that they remain stagnant and obstruct the minute capillaries. In this state of things it may happen that the serosity of the blood, being the thinnest and most liquid portion, impelled by the *vis a tergo,* is still able to traverse (at least partially) the obstructed vessels, and then exudes from them, possessed of those properties which *Hunter* has so faithfully described.

If from the violence of the inflammatory action the life of the part be destroyed, the process of putrefaction quickly begins, the capillary vessels gorged with the blood-globules burst and allow the globules, along with the decomposed serosity, to escape; in this manner we may explain the formation of the brown-coloured and turbid sanies which flows from a part affected with gangrene."—*Gazette Medicale.*

---

### MISCELLANEOUS NOTICES.

### 1. *State of the Ovaries during Menstruation.*

Some recent writers have alleged that each act of menstruation is accompanied with the rupture of one of the *Graafian* vesicles. In a case of very rapidly fatal fever, occurring in a young woman immediately after one of the catamenial periods, —reported by M. *Devay* in the L'Experience, No. 296—the following appearances were found on dissection. The outer surface of the right ovary was injected, and presented towards its upper edge a small lacerated opening, in which there lay a minute coagulum of blood that was still soft. On cutting through the substance of the ovary, there was observed another cicatricula of somewhat less recent date, and which contained a small sanguineous concretion of the size of a hemp-seed: three *Graafian* vesicles were counted.

The left ovary exhibited several scars of older date. The right Fallopian tube contained a little fluid blood; the left one was quite empty. Some sanguinolent mucus was observed on the inner surface of the uterus.

### 2. *French Letter Writing.*

Our neighbours seem always to be walking on stilts, when they approach even the very threshold of the Academy. Here is a letter from the "grande orthopœdiste," M. *Guerin,* to the President of this learned bcdy.

"Sir,—If I deemed the honour to be admitted as a candidate to a vacant seat in the Academy only as a glorious recompense, I should be more than satisfied with the favour, with which my first 'candidature' was received. But I feel that I better appreciate the true character of this distinction, by regarding it as the most illustrious consecration that can be bestowed on scientific works and ideas. It is from this consideration, Monsieur le President, that I am induced to offer myself as a candidate for the seat now vacant in the department of medicine and surgery.

"The Academy is aware that, for several years past, I have been engaged in researches undertaken under its inspiration, researches which have given rise to a great number of novel applications in surgery. I should, therefore, but ill comprehend its intentions, and be especially ungrateful for the encouragement which it has given to my labours, if I withdrew from a concours, where they may receive a new and powerful impulsion from its distinguished patronage. I take the liberty, therefore, to hope that the flattering reception which it condescended to give to my recent application, may be an additional inducement to examine with some interest and favour my titles in support of my ‘candidature’ in surgery.”

3. *Injection of Dropsical Joints, Cysts, &c.*

M. *Velpeau* announces that, for some years past, he has been engaged in preparing “un grand travail,” on the treatment of dropsical cysts by means of puncture and subsequent injection of the cavities with a solution of iodine. He has effected a cure in a number of cases of serous and sanguineous cysts in different parts of the body by this method. He has applied it with success in five cases of sacculated goitre, or hydrocele of the neck, as it has been called; and he has also tried it in a few cases of dropsy of the knee-joint.

At the date of his announcment, he had four patients, at La Charité hospital, in whom he had injected the iodine solution into this articular cavity: “leur etat est très satisfaisant.”

4. *Dog-killing for the Promotion of Science.*

We observe that M. *Bouvier*, one of the orthopœdist surgeons of Paris, has been entertaining the Academy with a report of some experiments which he had performed upon dogs, poor creatures, to ascertain the effects of dividing one or more of the tendons of the upper and lower extremities. In pointing out the application of the results of these experiments to the treatment of deformities, he very coolly characterises them (the experiments) as “a sort of researches which has the advantage of being followed soon afterwards by the microscopic examination of the parts, and which is not attended with the inconvenience of experimenting directly upon the human subject.”—Cruel and cowardly!

*Vegetable Morphology.*

It is pleasing to pass from the revolting subject last mentioned, to the ever gay and attractive scene of the vegetable world; in which experiments indeed may be performed without cruelty to other beings, or hardening of our own hearts.

“What can be more interesting,” says a recent French writer, “than the study of the metamorphoses which different parts of plants are subject to. Any one looking at the stamens of a rose, or of a ranunculus, would scarcely think of assimilating them with the leaves or petals of these plants; but if he examines the nymphæa or water-lily, he will be less surprised at being told that the one part (the stamen) is convertible into the other (the petal); for he will perceive, in the specimen before him, all the possible transitions between these two parts of the flower well characterised. Again, it is well known that excessive nourishment deprives many of the flowers of our gardens of their stamens, and causes them to change into elegant petals. If any doubt remained on this point, a most convincing proof of its reality is furnished by the Aquilegia or *Columbine.* Nothing can less resemble the spurred horns of its corolla than its small and slender stamens; yet, by cultivation, these latter are found to become metamorphosed into cornuted petals, which terminate in a spur; and, to prove the truth of this still more convincingly, we may often observe all the possible shades or degrees of transition between the stamen and the horn in one and the same flower.

“In the Canna, or Indian reed, we notice an equally striking illustration of the change; on one side there is often a simple unilocular anther to be seen, and on the other a petal in its place. .. .. .. .. .. We thus see that a stamen is nothing else but a metamorphosed petal; and as it (the petal) is only a variety of a leaf, the stamen can be regarded as nothing more. A leaf enfeebled becomes a petal; and more enfeebled still, it becomes a stamen."

6. *Mutual Friendship of distinguished Anatomists and Painters.*

It is well known that the great painters in the age of Leo X. devoted much of their attention to the study of anatomy. *Leonardi da Vinci* made the drawings for the Anthropotomia of *Della Torre*, and *Michael Angelo* those for the similar work of his friend *Colombus*. The prince of painters, the divine *Raffaelle di Urbino,* devoted much of his time to the composition of a work on human and comparative myology; and on the other hand, *Francesco Fresconi,* the physician of three successive Popes, was a most accomplished sculptor; and *Beranger de Carpi,* one of the earliest advocates of the use of mercurial fumigations in the treatment of syphilis, was so ardent an admirer of the fine arts that, on several occasions, he would accept of no other fee except a fine painting or statue from his patients. *Vasari* tells us that the Cardinal *Colonna* was obliged to give a celebrated painting by *Raphael* to the art-loving doctor, in payment for his professional aid.

*Titian,* with the assistance of one of his pupils, sketched the admirable drawings for the great work on anatomy by *Vesalius.* The great painter of Venice fully appreciated the merits of the young anatomist; for we find that, when he had finished the portraits of *Charles* V., *Francis* I., the Grand Signor *Soliman* and other crowned heads, he immediately commenced those of *Vesalius** and *Ariosto;* the aristocracy of talent appearing to him worthy of a place beside the aristocracy of rank.

7. *Important Academic Information.*

M. *Briquet* wrote to the Academy a month or two ago, announcing that quinine, in large doses, has the power of checking attacks of rheumatism, and intimating that he should soon send a memoir on the subject.

M. *Bouillaud* remarked, that rheumatism is usually a most rebellious disease, whatever mode of treatment be followed; and concluded by saying, that if M. *Briquet* can prove his assertion by well-established facts, he will render great service to science and humanity. M. *Martin-Solon* said that, according to his experience, no remedy is so efficacious as the nitrate of potash in very lare doses —as much as 10 drachms may be administered in 24 hours. Usually in five days or so, the disease may be thus *jugé.* He also promised to send a memoir to the Academy on the question. (How very instructive!)

8. *A Medical Poem.*

The French are fairly entitled to bear the bell from all other nations, as the legitimate children of *Apollo,* the god alike of poetry and of medicine. Where is the country that has produced so many sons of song among her doctors as "La Belle France?" Two or three years ago we heard of a poem on the very *pathetic* subject of (what do you think, reader?) *syphilis;* and behold, now we have one, in six cantos, entitled "Code Moral du Medecin."

The present bard, Dr. *Andrevetan,* has the merit, if he cannot lay claim to any other, of having enriched the medical vocabulary with some choice phrases.

A few examples will suffice. In place of the common and every-day name of doctors, we are called *ministers of health, children of Chiron, disciples of the Centaur, sons of Lucina, neophytes of the god of Cos, priests of Hygeia, of Isis, and of Æsculapius, pontiffs of Apis and Osiris, favorites of Zelesphorus, children*

---

* This celebrated portrait was bequeathed by Baron *Portal* to the Academy of Medicine, and is now hung up in their hall, immediately behind the president's chair.

*of Apollo, successors of Atothis, &c. &c. !!* So much for the *medecins;* now for the profession, *la medecine.* Sometimes it is called *the altar of the god of Epidaurus,* or the *santuary of Apollo;* at other times it becomes instinct with life and springs forth as the goddess *Panacea,* or *the Divine Hygeia;* or lastly, it is *the offspring of Phœbus,* or the *handmaid of Lucina.* The same richness of language pervades the work on other subjects. Chemistry is the *art of Hermes;* man is *the son of Japhet;* death is *the Daughter of Night,* or *Atropos;* fire is *the gift of Vulcan;* wine is *the nectar of Bacchus;* men of science are *the favourites of Minerva,* and soldiers are *the children of Mars.* Even the names of the present nations in Europe are raised to classical honours; thus the French are the *Gauls,* the Allemands are *the Germans,* England is always, " comme de juste" (!) *perfidious Albion,* Spain is *Iberia,* and Sicily recovers its old name of *Trinacria.*

9. *Vitalism.*

On the occasion of a recent account of a fatal case in the St. Louis hospital, where the reporter alludes to the " insignificance decourageante des alterations anatomiques" found on dissection, M. *Cayol* makes the following observations: " *Discouraging!*—We should register this word, this naive expression of the state of science, this cry of distress from anatomism, a cry which at length is heard to issue from all the benches of the school. We have recently seen two physicians of eminence, at the head of two great hospitals, become *discouraged* in presence of an epidemic, (that of *cerebro-spinitis* alluded to in a former number) the character and curative indications of which were alike mysterious to them; and now a distinguished pupil of the same school experiences a similar *discouragement* in the presence of a corpse, because he does not find on dissection what he fully expected with all the ardour and good faith of his age. The case consequently appears to him incomplete: for he has been taught to believe that the existence of the disease necessarily indicates such and such anatomical lesions to be present. Now we ask, would not this gentleman have formed a much more rational idea on the case in question, and been much more satisfied with the appearances found on dissection, if he had been instructed that the *disease is a vital act,* that it ceases with life, and that the necroscopic alterations—the importance of which we are far from denying where they really exist—are only the effects and results (not however the necessary ones) of this vital act, this abnormal function, which constitutes the disease? We cannot too often repeat this philosophic formula of vitalism, which is, in our opinion the very key of pathology."

10. *Hernia, Operation in.*

M. *Sedillot,* in commenting on a fatal case of hernia in his lectures, recalled to his hearers' attention the important saying of Sir *A. Cooper*—le grand chirurgien dont la pratique etait immense et le jugement si droit—" that if he were himself the subject of a strangulated hernia, he should not wish twelve hours to pass over without an operation."

*Paré, Le Blanc,* and many other celebrated surgeons have given a similar advice.

11. *Diagnosis of Scabies.*

M. *Sedillot,* after describing the mode of detecting the acarus and its appearances, says; " It is not however the presence of this insect that is to be considered as the distinctive sign of the disease; for it is often absent altogether; and even when not, it is by no means easily found by the inexperienced; but *it is the groove annexed to and leading from the papulæ*—a phenomenon which is constant in a certain number, whatever be their state of dessication. As this appearance does not exist in prurigo, its presence enables us " de formuler un jugement precis."

12. *Purulent Infection.*

M. *Sedillot* adopts, in reference to the ætiology of this most dangerous cachexy, the opinion that was first propounded by M. *Velpeau,* viz. that it depends upon the direct admixture of purulent matter with the blood, and the consequent contamination of the vital fluid.* There is not *necessarily* any phlebitis present, as alleged by M. *Blandin;* although certainly this lesion is of frequent occurrence. The theory of M. *Tessier* is utterly inadmissible: "Is it possible," asks the professor of Strasbourg, "to separate two facts so well linked together, as a pre-existent suppuration, and a consecutive purulent infection?"

13. *M. Roux's Hospital Practice: Lithotomy and Lithotrity.*

During the five years from 1836 to 1841, 24 patients have been operated upon for urinary calculus in the Hôtel Dieu; in six cases by lithotrity and in 18 by lithotomy. In all the first set, the patients recovered; but of the 18 who were cut, seven died.

In 1841, seven calculous patients were admitted under the care of M. *Roux:* in one case, that of a young girl 11 years of age, death took place on the very day of her admission, in consequence of a profuse diarrhœa, which had utterly wasted her strength. Of the other six patients, four were treated by lithotomy and two by lithotrity: of the entire number only one—one of the four—recovered. In one unfortunate case of lithotrity, the *brise-pierre* instrument had formed a false passage about the neck of the bladder. (Frightful mortality on the whole.)

14. *Acupuncture in Neuralgia.*

M. *Lallemand* of Montpelier has for many years been in the habit of using acupuncture in cases of *genuine* neuralgia with very decided benefit: against rheumatic pains, he says, it is quite inefficacious. We must therefore be careful in discriminating the cases for its employment; otherwise we shall certainly be disappointed. If the pain be limited to the *trajet* of the nerves, we may with tolerable confidence promise relief, if not a complete cure, of the suffering. M. *Lallemand* relates many cases; one we shall briefly notice. A man had for six months been afflicted with most severe pain along the whole course of the sciatic nerve; five needles were inserted along its tract, and left in for three hours. The application was repeated at intervals of one or two days, four successive times; and the man was then completely cured.

15. *Importance of Veterinary Medicine.*

At the recent annual meeting at Strasbourg of the Scientific Association of France, M. *Falk* read a very sensible paper with the view of shewing the importance of medical men making themselves acquainted with veterinary medicine. The utter neglect of this study is certainly to be regretted, as some valuable hints for the treatment of diseases in the human subject might be derived from what may be called comparative pathology. Every one in the present day recognises the importance of a knowledge of the anatomy of the lower animals;—why not then of their diseases?

The suggestion of M. *Falk* was well received by the Association.

---

* There is a difficulty which this theory does not solve: how is it that we daily see cases of purulent deposits being quickly absorbed without inconvenience to the system, or a single symptom of constitutional *infection?* Query. Has an electrical or galvanic agency any thing to do in such a case?

